# Cytotoxic Activity of Novel GnRH Analogs Conjugated with Mitoxantrone in Ovarian Cancer Cells

**DOI:** 10.3390/molecules29174127

**Published:** 2024-08-30

**Authors:** Christos Markatos, Georgia Biniari, Oleg G. Chepurny, Vlasios Karageorgos, Nikos Tsakalakis, Georgios Komontachakis, Zacharenia Vlata, Maria Venihaki, George G. Holz, Theodore Tselios, George Liapakis

**Affiliations:** 1 Department of Pharmacology, School of Medicine, University of Crete, 70013 Heraklion, Greece; xristosmarkatos@gmail.com (C.M.); bkarageorgos@hotmail.com (V.K.); chem2763@edu.chemistry.uoc.gr (N.T.); chem2785@edu.chemistry.uoc.gr (G.K.); 2 Department of Chemistry, University of Patras, 26504 Rion, Greece; georgiabiniari96@gmail.com; 3 Department of Medicine, State University of New York (SUNY), Upstate Medical University, Syracuse, NY 13210, USA; chepurno@upstate.edu; 4 Flow Cytometry Facility, Institute of Molecular Biology and Biotechnology of the Foundation for Research and Technology Hellas (IMBB-FORTH), 70013 Heraklion, Greece; xvlata@imbb.forth.gr; 5 Department of Clinical Chemistry, School of Medicine, University of Crete, 71003 Heraklion, Greece; venycham@uoc.gr; 6 Department of Medicine and Pharmacology, State University of New York (SUNY), Upstate Medical University, Syracuse, NY 13210, USA; holzg@upstate.edu

**Keywords:** gonadotropin-releasing hormone, receptor, mitoxantrone, ovarian cancer, cytotoxic drugs

## Abstract

The gonadotropin-releasing hormone (GnRH) receptor (GnRH-R) is highly expressed in ovarian cancer cells (OCC), and it is an important molecular target for cancer therapeutics. To develop a new class of drugs targeting OCC, we designed and synthesized Con-3 and Con-7 which are novel high-affinity GnRH-R agonists, covalently coupled through a disulfide bond to the DNA synthesis inhibitor mitoxantrone. We hypothesized that Con-3 and Con-7 binding to the GnRH-R of OCC would expose the conjugated mitoxantrone to the cellular thioredoxin, which reduces the disulfide bond of Con-3 and Con-7. The subsequent release of mitoxantrone leads to its intracellular accumulation, thus exerting its cytotoxic effects. To test this hypothesis, we determined the cytotoxic effects of Con-3 and Con-7 using the SKOV-3 human OCC. Treatment with Con-3 and Con-7, but not with their unconjugated GnRH counterparts, resulted in the accumulation of mitoxantrone within the SKOV-3 cells, increased their apoptosis, and reduced their proliferation, in a dose- and time-dependent manner, with half-maximal inhibitory concentrations of 0.6–0.9 µM. It is concluded that Con-3 and Con-7 act as cytotoxic “prodrugs” in which mitoxantrone is delivered in a GnRH-R-specific manner and constitute a new class of lead compounds for use as anticancer drugs targeting ovarian tumors.

## 1. Introduction

The Gonadotropin-Releasing Hormone GnRH is a decapeptide that controls the pulsatile release of the gonadotropin hormones luteinizing hormone (LH) and follicle-stimulating hormone (FSH) from the pituitary gland [1]. FSH and LH in turn regulate the function of the reproductive system, including the synthesis of estrogen and progesterone in females. GnRH exerts its actions through interacting with GnRH-specific receptors (GnRH-R) which belong to the family of G-protein coupled receptors (GPCRs) [1,2]. The binding of GnRH to GnRH-R results in receptor activation and the subsequent stimulation of Gq proteins and intracellular calcium mobilization [1]. In addition to Gq proteins, the GnRH-R stimulates Gi-proteins [1].

Apart from the physiological expression of the GnRH-R in the pituitary and reproductive organs such as myometrium or ovaries, GnRH and its receptors are also detected in cancer cells, including the ovarian ones, leading to the speculation that GnRH plays an autocrine/paracrine role in these cells, regulating proliferation, cell cycle and apoptosis [3,4,5,6,7,8,9,10,11,12]. Ovarian cancer is the seventh most-common cancer among women [13], with an age-standardized incidence of 6.7 per 100,000. Ovarian cancer accounts for an estimated cumulative mortality risk of 0.46%, with the epithelial type being the most common one, accounting for 90% of all cases [14,15]. This is because epithelial ovarian cancer is diagnosed in the advanced stages, whereas early stages do not have noticeable symptoms [15]. Ovarian cancer is hormone-dependent, promoted by estrogens and FSH [16,17,18,19].

The sustained administration of GnRH agonists, such as leuprolide, results in the inhibition of the secretion of gonadotropins and subsequent suppression of their actions in the ovary, through various mechanisms, including the down-regulation of the GnRH-R [8,20,21]. Based on this property, several GnRH agonists have been used to treat various ovarian cancers [3,20]. GnRH agonists also decrease the proliferation and promote the apoptosis of ovarian cancer cells, including the SKOV-3 cells used in this study, directly by interacting with GnRH receptors expressed in these cells [3,10,11,22,23,24,25,26,27,28,29,30]. These properties render GnRH agonists as promising anticancer drugs [8]. The cytotoxic effects of GnRH analogs on ovarian cancer cells were mainly associated with the GnRH-R-mediated stimulation of the Gi proteins, which resulted in the activation of a protein phosphatase, and the subsequent decrease in the phosphorylation of EGF receptors [31]. In contrast, synthesis and the secretion of gonadotropins in the pituitary are attributed to the GnRH-R-mediated activation of Gq proteins [3]. 

**Figure 1:**
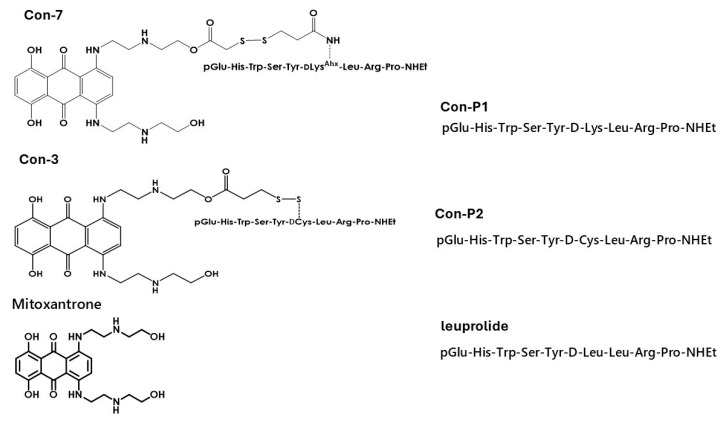
Chemical structures of the GnRH conjugates, Con-3 and Con-7, and the unconjugated peptides, Con-P2 and Con-P1.

A different therapeutic approach against GnRH-dependent cancers is the GnRH-R-targeted therapy. Specifically, anticancer GnRH conjugates have been developed via linking through an ester bond, [D-Lys^6^] GnRH, with cytotoxic doxorubicin or its derivatives [12,32,33,34,35,36]. One of these analogs is called AN-152. AN-152 exerts its cytotoxic actions through its interaction with the GnRH-R in tumor cells, the internalization of the receptor–conjugate complex, and the subsequent intracellular release of doxorubicin. A drawback of this approach is the toxicity of doxorubicin and the vulnerability of these conjugates to plasma carboxylesterases, which cleave the ester bond between GnRH and doxorubicin, thus releasing the latter before reaching the tumors [33,34,35]. In the present study, we characterized the cytotoxic properties of the GnRH conjugates, Con-3 and Con-7, which have been created by our research team by linking modified leuprolide analogs with the less toxic anticancer agent, mitoxantrone, through a disulfide bond [37,38,39]. Specifically, mitoxantrone causes significantly less nausea, vomiting, diarrhea, anorexia, stomatitis, and alopecia and appears to be less cardiotoxic than doxorubicin [37,38]. The modified leuprolide analogs, which were used to create the Con-3 and Con-7, by linking them to mitoxantrone, are nine amino-acid peptides created by our research team and named Con-P2 and Con-P1, respectively (Figure 1). Specific, the Con-P1 and Con-P2 correspond to analogs 4 and 2 of our previous study, which were analytically described in the Supplementary Materials of this study [39].

## 2. Results

### 2.1. Con-3 and Con-7 Are GnRH-R Agonists

**Figure 2:**
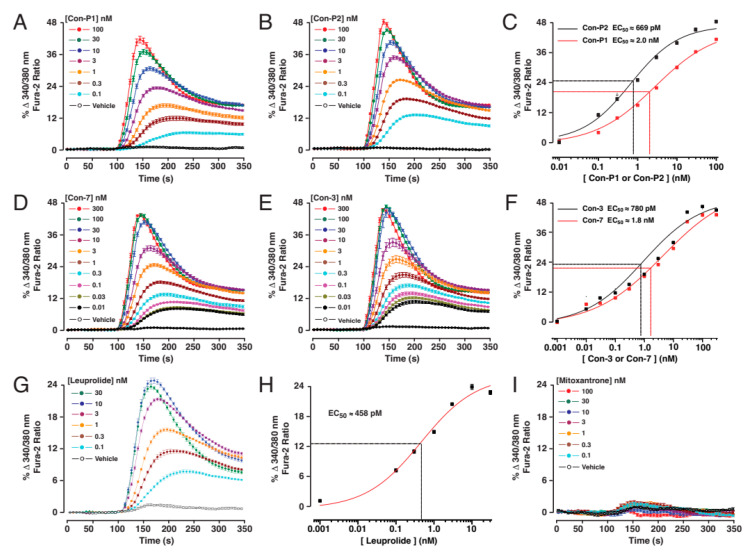
Real-time kinetic assays of GnRH-R agonist action in HEK293 cells. HEK293 cells transfected with the human GnRH-R served as a platform with which to test for agonist actions of individual test compounds. These cells were loaded with the ratiometric Ca^2+^ indicator fura-2 so that the change in intracellular [Ca^2+^] in response to agonist stimulation could be monitored in real time in a 96-well format. For these assays, the individual test compounds were administered at the 100 s time point. Note that the y-axis values are expressed as the % 340/380 nm fura-2 emission ratio in which an increase in the ratio signifies an increase of [Ca^2+^]. Concentration–response studies demonstrated the Ca^2+^-elevating agonist properties and EC_50_ values for Con-P1 and Con-P2 (**A**–**C**) or Con-7 and Con-3 (**D**–**F**). Leuprolide also acted as a GnRH-R agonist (**G**,**H**), whereas unconjugated mitoxantrone was without effect (**I**).

To test for the GnRH-R agonist actions of the individual test compounds, we monitored the characteristic increase in intracellular [Ca^2+^] that results from receptor stimulation. For this purpose, fura-2-based kinetic assays to monitor the [Ca^2+^] were performed in real-time using suspensions of HEK293 cells transiently transfected with the human GnRH-R (Figure 2A–I). This analysis demonstrated that the free (unconjugated to mitoxantrone) peptides Con-P1 and Con-P2 raised the levels of [Ca^2+^] in a concentration-dependent manner, thereby establishing the EC_50_ values of 2 nM and 0.67 nM for Con-P1 and Con-P2, respectively (Figure 2A–C). Using this same approach, the EC_50_ values for Con-7 and Con-3 were established to be 1.8 nM and 0.78 nM, respectively (Figure 2D–F). Control experiments confirmed that the GnRH-R agonist leuprolide (EC_50_ 0.46 nM) also exerted an agonist action (Figure 2G,H), whereas unconjugated mitoxantrone was without effect (Figure 2I).

### 2.2. Con-3 and Con-7 Decrease the Proliferation of SKOV-3 Cells

**Figure 3:**
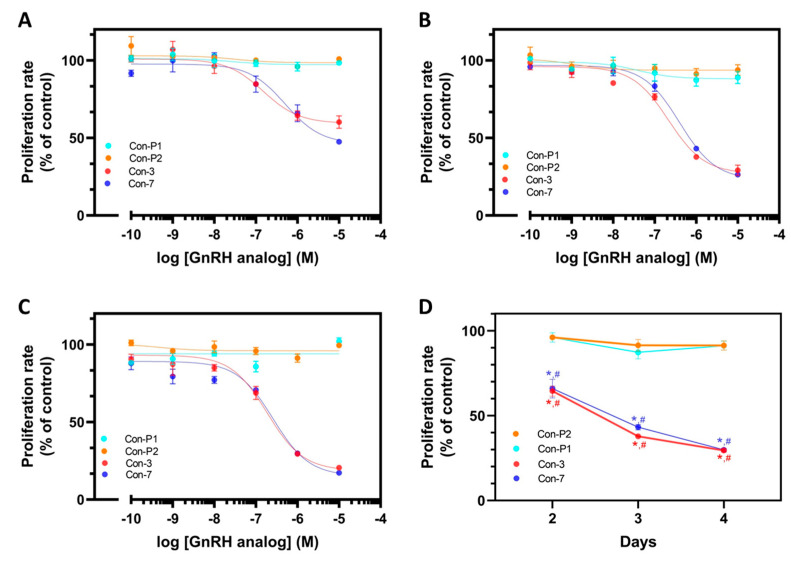
Antiproliferative activity of the GnRH analogs. Decrease in the proliferation rate of the SKOV3 cells after their treatment with increasing concentrations of Con-3, Con-7, Con-P1 or Con-P2 for 2 days (**A**), 3 days (**B**) and 4 days (**C**) at 37 °C. At the end of the incubation, the cells were further incubated with 0.5 mg/mL MTT for 4 h at room temperature and the formed purple formazan crystals were quantified by measuring absorbance at 594 nm. The means ± S.E (triplicate determination) are shown from a representative experiment performed 3 times with similar results. The proliferation rates of cells treated with 1 μΜ of GnRH analogs were used to plot the graph D which depicts the decrease in cell growth over the incubation time (**D**). Statistical analysis was performed using one-way ANOVA. The asterisk symbols indicate the proliferation rates of cells treated with Con-3 or Con-7 which were significantly different from that of untreated cells (controls) (*p* ˂ 0.05). The pound symbols indicate proliferation rates of cells treated with Con-3 or Con-7 which were significantly different from those treated with Con-P2 or Con-P1, respectively (*p* ˂ 0.05).

The antiproliferative effects of GnRH-mitoxantrone conjugates, Con-3, and Con-7, were evaluated using the SKOV-3 cells. The Con-3 and Con-7 conjugates decreased the proliferation rate of SKOV-3 cells in a dose-dependent manner on days 2, 3, and 4 (Figure 3A–C). The half-maximal inhibitory concentrations (IC_50_) of Con-3 on days 2, 3 and 4 were 0.90 ± 0.70 μΜ, 0.80 ± 0.42 μΜ and 0.80 ± 0.42 μΜ, respectively (*n* = 3 experiments), whereas the corresponding ones of Con-7 were 0.68 ± 0.07 μΜ, 0.88 ± 0.17 μΜ and 0.60 ± 0.20 μΜ (*n* = five experiments). 

To demonstrate the importance of the incubation time on cell growth, we presented the cell proliferation data as a graph by plotting the time (days 2–4) versus the percentage of the proliferation rate of cells after their treatment with Con-3 or Con-7 at a concentration of 1 μM, which is the closest value to the IC_50_ of these conjugates (Figure 3D). In marked contrast, Con-P1 and Con-P2 did not affect the proliferation of SKOV-3 cells over time and at various concentrations (Figure 3A–D). 

**Figure 4:**
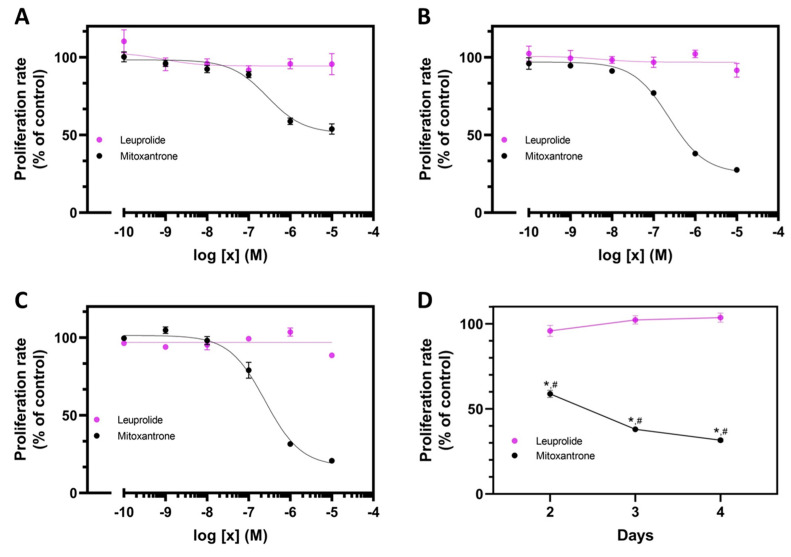
Antiproliferative activity of the mitoxantrone or leuprolide. Decrease in the proliferation rate of the SKOV-3 cells after their treatment with increasing concentrations of mitoxantrone or leuprolide for 2 days (**A**), 3 days (**B**) and 4 days (**C**) at 37 °C. At the end of the incubation, the cells were further incubated with 0.5 mg/mL MTT for 4 h at room temperature and the formed purple formazan crystals were quantified by measuring absorbance at 594 nm. The means ± S.E (triplicate determination) are shown from a representative experiment performed 3 times with similar results. The proliferation rates of cells treated with 1 μΜ of mitoxantrone or leuprolide were used to plot the graph D which depicts the decrease in cell growth over the incubation time (**D**). Statistical analysis was performed using one-way ANOVA. The asterisk symbols (*) indicate the proliferation rates of cells treated with mitoxantrone which were significantly different from that of untreated cells (controls) (*p* ˂ 0.05). The pound symbols (#) indicate the proliferation rates of cells treated with mitoxantrone which were significantly different from those treated with leuprolide (*p* ˂ 0.05).

As Con-3 and Con-7, the mitoxantrone decreased the proliferation rate of SKOV-3 cells in a dose-dependent manner, with IC_50_ values 0.48 ± 0.21 μΜ, 0.42 ± 0.09 μΜ and 0.28 ± 0.06 μΜ, on days 2, 3 and 4, respectively (*n* = 3 experiments) (Figure 4A–C). Similar to the GnRH conjugates, the antiproliferative effects of mitoxantrone were time-dependent (Figure 4D). In marked contrast, the leuprolide, as Con-P1 and Con-P2, did not affect the proliferation of SKOV-3 cells over time and at various concentrations (Figure 4A–D). 

### 2.3. Con-3 and Con-7 Induce Apoptosis in SKOV-3 Cells

**Figure 5:**
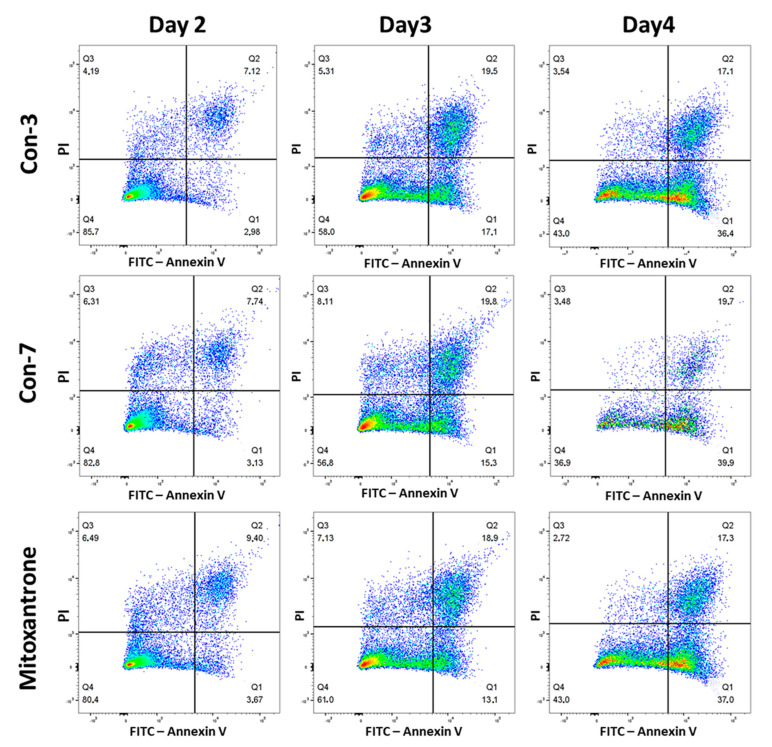
Determination of viability, apoptosis, or necrosis of SKOV-3 cells after their treatment with 1 μM Con-3, Con-7, or mitoxantrone for 2, 3, and 4 days at 37 °C. At the end of the incubation the cells were further incubated in binding buffer supplied by Biotium, (CF488A Apoptosis Detection kit) and analyzed on a flow cytometer (excitation 488 nm), in the FITC channel (filter 530 nm) for detection of the Annexin V-positive cellsand in the propidium iodide, or PI, channel (filter 616 nm) for detection of the PI-positive cells. The figures are from a representative experiment performed three times with similar results. The mean ± SEM (Standard Error of the Mean) values of apoptotic cells are shown in Table 1. A higher resolution of the images in Figure 5 is provided in the Supplementary Material (Figures S1–S9).

We next evaluate the abilities of Con-3 and Con-7 at concentrations of 1 μΜ to induce cell death by determining the percentages of live, apoptotic and necrotic cells after treatment of SKOV-3 cells with the GnRH conjugates. As shown in Figure 5 and Figures S1–S3 on day 2, the majority of SKOV-3 cells were viable (Q4 quadrant), treated either with Con-3, Con-7 or mitoxantrone, whereas the percentage of apoptotic cells (late or early apoptotic) (Q1, Q2 quadrants) was only 10.1%, 10.87% and 13.1% after treatment with Con-3, Con-7 or mitoxantrone, respectively. On day 3, the number of viable cells decreased with a concomitant increase in the percentage of apoptotic cells, which was 36.6%, 35.1% and 32% after treatment with Con-3, Con-7 or mitoxantrone, respectively (Figure 5 and Figures S4–S6). Cell viability further decreased on day 4 with a concomitant increase in apoptosis. Specifically, the percentage of apoptotic cells was 53.5%, 59.6% and 54.3% after treatment with Con-3, Con-7 or mitoxantrone (Figure 5 and Figures S7–S9). In all phases, the percentage of the necrotic and late apoptotic cells (Q3 quadrant) was very small.

Similar to their effects on cell proliferation, the mitoxantrone-lacking peptides, leuprolide, Con-P1 and Con-P2, did not affect the viability of SKOV-3 cells (Figure 6 and Figures S10–S21).

**Figure 7:**
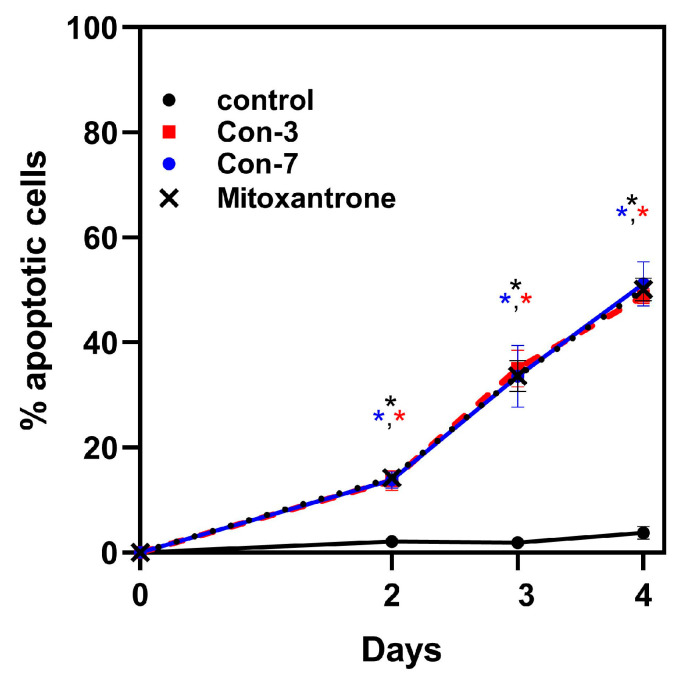
Percentages of apoptotic SKOV-3 cells after their treatment without (untreated) or with 1 μM of Con-3, Con-7 or mitoxantrone for 2, 3, or 4 days. The means ± SEM (shown in Table 1) are from three independent experiments. Statistical analysis was performed using repeated measures ANOVA. The asterisks indicate that the percentage of apoptotic cells treated with the Con-3, Con-7 or mitoxantrone is significantly higher (*p* < 0.01) than that of untreated cells (control).

To demonstrate the importance of incubation time on cell apoptosis, we presented the flow cytometry data as a graph by plotting the time (days 2–4) versus the percentage of apoptotic cells after their treatment with Con-3, Con-7, or mitoxantrone at a concentration of 1 μM (Figure 7). This graph clearly demonstrates that the incubation time is a crucial parameter for cell viability because its extension from two to four days was associated with a significant increase in the percentage of apoptotic cells treated with the Con-3, Con-7 or mitoxantrone, compared to untreated cells, as shown in Table 1. In contrast, the incubation time did not alter the viability of the untreated cells (Figure 7). 

**Figure 8:**
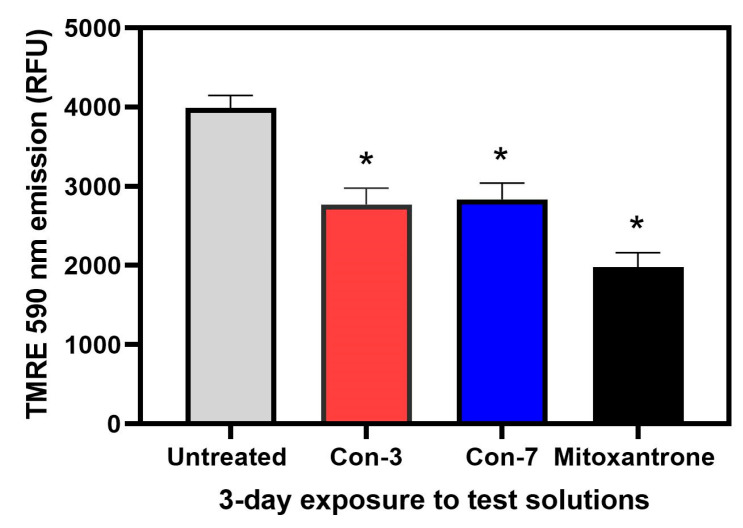
TMRE-based detection of mitochondrial membrane potential (ΔΨm) of SKOV-3 cells. The change in ΔΨm was detected using TMRE after treatment of SKOV3 cells with 1μM Con-3, Con-7 or mitoxantrone for 72 h. The TMRE fluorescence intensities are represented as histogram bars for results obtained in three independent experiments. Con-3, Con-7 and mitoxantrone-treated cells displayed significantly reduced fluorescence as compared with untreated cells, indicating loss of mitochondrial membrane potential. The data are expressed as the mean ± SEM. The asterisks indicate that the TMRE fluorescence intensities of cells treated with the Con-3, Con-7, or mitoxantrone are significantly lower (*p* < 0.01; post hoc Bonferroni analysis, ANOVA) than that of untreated cells (control).

The apoptosis of SKOV3 cells by Con-3, Con-7 and mitoxantrone was further validated in a mitochondrial membrane potential (ΔΨm) assay, which utilizes the cell-permeable, cationic dye, tetramethylrhodamine ethyl ester (TMRE) to detect the ΔΨm. TMRE accumulates in healthy mitochondria with polarized membrane, but not in the mitochondria of apoptotic cells. Mitochondrial permeabilization and the loss of the mitochondrial transmembrane potential are associated with cell apoptosis and a drop in fluorescence of TMRE [40]. As shown in Figure 8, treatment of SKOV3 cells with Con-3, Con-7 or mitoxantrone for 3 days significantly reduced the accumulation of the TMRE and thus its fluorescence, indicating loss of mitochondrial membrane potential.

### 2.4. Intracellular Accumulation of Mitoxantrone after Treatment of SKOV-3 Cells with Con-3 or Con-7

**Figure 9:**
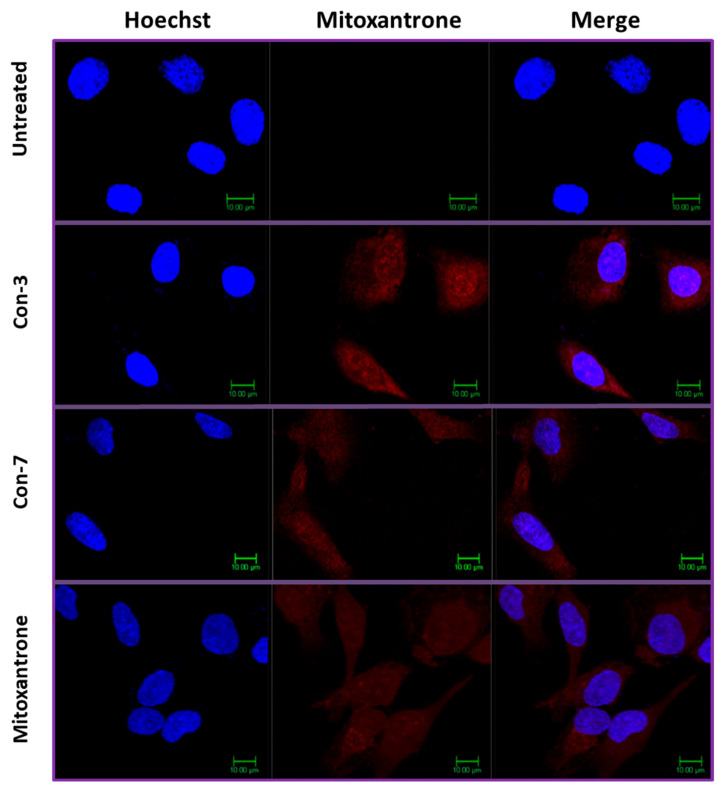
Confocal microscopy images of SKOV-3 cells treated with or without the GnRH-mitoxantrone conjugates, Con-3, Con-7 or mitoxantrone at a concentration of 1 μΜ for 6 h at 37 °C. At the end of the incubation, the cell nuclei were stained with the DNA-staining Hoechst 33342 dye and confocal fluorescent images were obtained after sequential excitation at 350 nm (for the Hoechst dye) and 638 nm (for the mitoxantrone). The emission filters of 461 nm and 650 nm–700 nm were used for collecting blue (Hoechst dye) and red (mitoxantrone) signals in separate channels. The left images show the nuclei staining with Hoechst dye (blue), whereas the middle ones depict the mitoxantrone fluorescence (red). The right images were created by merging the right and middle ones and depict the localization of mitoxantrone (red) in both the cytoplasm and the nuclei. The figures are shown from a representative experiment performed three times with similar results.

To determine the accumulation of mitoxantrone within the SKOV-3 cells, we used confocal microscopy, exploiting the ability of mitoxantrone to fluoresce [41]. Specifically, the excitation maxima of mitoxantrone are at 610 and 660 nm, whereas its emission maximum is at 685 nm. To stain the nuclei, we used the Hoechst dye [42]. To demonstrate the nuclear localization of mitoxantrone, we merged the images showing the localization of Hoechst dye with those depicting the mitoxantrone fluorescence. As shown in Figure 9, mitoxantrone fluorescence is detected in both the cytoplasm and nucleus of the SKOV-3 cells after 6 h treatment with 1 μM of Con-3, Con-7 or mitoxantrone. In contrast, the fluorescence signal was absent in untreated cells. Interestingly, the fluorescence intensities, and therefore the intracellular concentrations, of the free mitoxantrone and the conjugated one with the GnRH analogs in Con-3 and Con-7 were similar (Figure 9), as confirmed by the quantification of their fluorescence intensities using Image J software (1.54j, U.S. National Institutes of Health, Bethesda, MD, USA)and *t*-test statistical analysis of the results (Table 2).

### 2.5. Cisplatin Inhibits the Intracellular Accumulation of Mitoxantrone of Con-3 and Con-7 

**Figure 10:**
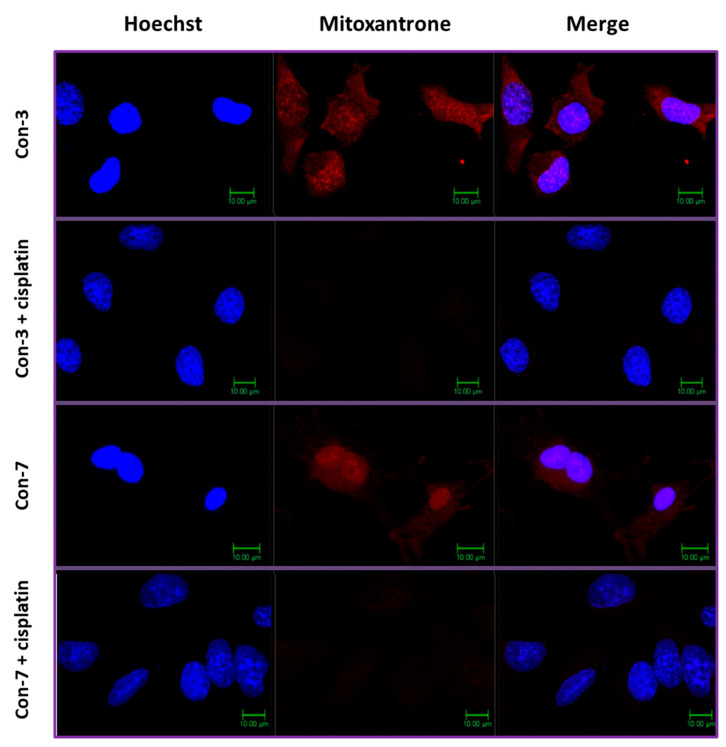
Confocal microscopy images of SKOV-3 cells treated with the GnRH-mitoxantrone conjugates, Con-3 or Con-7 (1 μΜ for 6 h) at 37 °C, in the presence or absence of cisplatin. Cisplatin was added to the cells 1 h before their incubation with the GnRH-mitoxantrone conjugates. At the end of the incubation, the cell nuclei were stained with the DNA-staining Hoechst 33342 dye and confocal fluorescent images were obtained after sequential excitation at 350 nm (for the Hoechst dye) and 638 nm (for the mitoxantrone). The emission filters of 461 nm and 650 nm–700 nm were used for collecting blue (Hoechst dye) and red (mitoxantrone) signals in separate channels. The left images show the nuclei staining with Hoechst dye (blue), whereas the middle ones depict the mitoxantrone fluorescence (red). The right images were created by merging the right and middle ones and depict the localization of mitoxantrone (red) in both the cytoplasm and the nuclei. The figures are shown from a representative experiment performed three times with similar results.

We hypothesized that the GnRH-conjugated mitoxantrone of Con-3 and Con-7 entered the SKOV-3 cells after the reduction in the disulfide bond linking the GnRH analogs with mitoxantrone and subsequent release of the latter from the conjugates. This chemical reaction could be accomplished by the thioredoxin system, which is overexpressed in cancer cells and reduces the disulfide bonds of proteins and peptides [43,44,45]. To verify this hypothesis, we incubated the SKOV-3 cells with cisplatin at a concentration of 30 μg/mL for 1 h before treating them with Con-3 or Con-7 conjugates. Cisplatin is a potent inhibitor of the thioredoxin system [46,47]. As shown in Figure 10, cisplatin abolished the accumulation of mitoxantrone and thus its fluorescence red signal within SKOV-3 cells treated with Con-3 (image Con-3 + cisplatin) or Con-7 (image Con-7 + cisplatin). 

**Figure 11:**
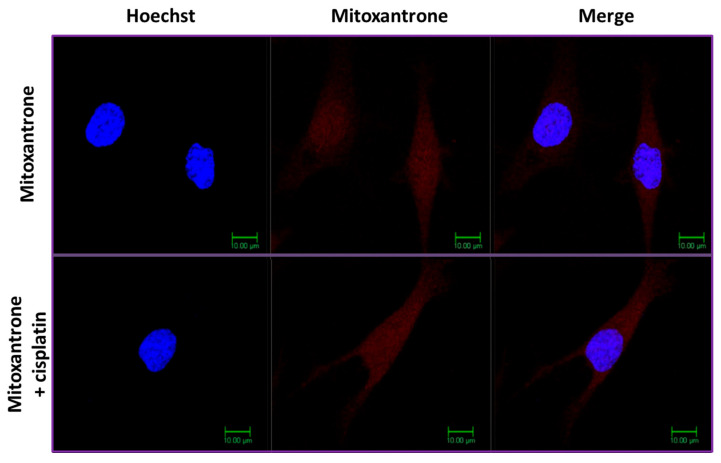
Confocal microscopy images of SKOV-3 cells treated with mitoxantrone (1 μΜ for 6 h) at 37 °C, in the presence or absence of cisplatin. Cisplatin was added to the cells 1 h before their incubation with mitoxantrone. At the end of the incubation, the cell nuclei were stained with the DNA-staining Hoechst 33342 dye and confocal fluorescent images were obtained after sequential excitation at 350 nm (for the Hoechst dye) and 638 nm (for the mitoxantrone). The emission filters of 461 nm and 650 nm–700 nm were used for collecting blue (Hoechst dye) and red (mitoxantrone) signals in separate channels. The left images show the nuclei staining with Hoechst dye (blue), whereas the middle ones depict the mitoxantrone fluorescence (red). The right images were created by merging the right and middle ones and depict the localization of mitoxantrone (red) in both the cytoplasm and the nuclei. The figures are shown from a representative experiment performed three times with similar results.

In marked contrast, cisplatin did not affect the intracellular accumulation of the free unconjugated mitoxantrone, which as a lipophilic drug passes freely through the plasma membrane of the cells (Figure 11). 

## 3. Discussion

In this study, we characterized the cytotoxic effects of the two novel GnRH conjugates, Con-3 and Con-7, on the SKOV-3 cells. The Con-3 and Con-7 conjugates bind to GnRH-R with high nanomolar affinity and have been created by linking the leuprolide-modified analogs Con-P1 and Con-P2 to the cytotoxic mitoxantrone through a disulfide bond [39]. We hypothesized that the presence of a GnRH analog to these conjugates serves as a guide that specifically delivers mitoxantrone to GnRH-R-expressing cancer cells, where it exerts its cytotoxic actions. Indeed, the Con-3 and Con-7 exerted antiproliferative effects on SKOV-3 cells by reducing their rate of proliferation to a similar extent to that observed after mitoxantrone treatment. Specifically, the antiproliferative actions of Con-3 and Con-7 were dose-dependent, displaying half-maximal inhibitory concentrations (IC_50_) of 0.6–0.9 µM on the second, third and fourth days of treatment. To determine whether the inhibition of proliferation of the SKOV-3 cells by Con-3 and Con-7 was associated with cell death, we monitored the induction and progression of cell apoptosis using flow cytometry and the markers annexin V and propidium iodide. Flow cytometric analysis revealed that SKOV-3 cells treated with Con-3, Con-7 or mitoxantrone, underwent cell death mainly via apoptosis and less through necrosis. The percentage of the apoptotic SKOV-3 cells largely increased over time, suggesting that the Con-3, Con-7 or mitoxantrone induced time-dependent apoptosis. The apoptosis of SKOV3 cells by Con-3, Con-7 and mitoxantrone was further validated in a mitochondrial membrane potential (ΔΨm) assay, which detects the apoptosis-associated loss of the mitochondrial transmembrane potential [40]. A treatment of SKOV3 cells with Con-3, Con-7 or mitoxantrone for 3 days significantly reduced the ΔΨm, in agreement with the apoptotic effect of these compounds detected in the Annexin V assay.

Interestingly, the inhibition of the proliferation rate was not in agreement with the percentage of apoptotic cells. For example, the percentage of the proliferation rate after two days of treatment with 1 μΜ of Con-3 was 64.4%, whereas the percentage of apoptotic cells was only 13.7%. Thus, despite the large reduction in the number of viable SKOV3 cells, Con-3 displayed weak apoptosis-associated changes as determined using annexin V and propidium iodide. Similarly, topotecan significantly decreased the number of viable cells and displayed a small apoptotic effect [48]. This discrepancy is likely due to the fact that several metabolic enzymes retain their activity during the induction of apoptosis, so the viable cells detected by the MTT assay might include a fraction of cells in early apoptosis which is reversible, as previously suggested [48]. Another factor that could contribute to this discrepancy is the determination of two different processes, cell viability in the MTT assay, and cell death in the Annexin V assay [49]. Cell viability is inferred by the evaluation of its metabolic activity, and it is detected by the MTT dye which is reduced into the insoluble formazan by the activity of NAD(P)H-dependent cellular oxidoreductases of viable cells. In contrast, the determination of cell death by Annexin V is based on the apoptosis-associated expression of phosphatidylserine on the surface of apoptotic cells. 

The antiproliferative and apoptotic effects of Con-3 and Con-7 were similar to those of the free unconjugated mitoxantrone, which displayed IC_50_ values of 0.28 µM–0.48 µM on the second, third and fourth days of treatment, and were comparable to those in different cancer cells [50]. In marked contrast, leuprolide and the free unconjugated GnRH analogs Con-P1 and Con-P2 did not exert apoptotic and antiproliferative effects on SKOV-3 cells. These results suggest that the cytotoxic effects of the GnRH conjugates were not due to their peptidic ingredients but rather exclusive to mitoxantrone. The mechanisms underlying the cytotoxic effects of mitoxantrone are well-studied [50,51,52]. Further support of this hypothesis is provided by our confocal microscopy experiments, which showed that, as the free unconjugated mitoxantrone, its conjugated form in Con-3 and Con-7 accumulated into the cytoplasm and nucleus of SKOV-3 cells, after its release from the GnRH conjugates. This finding leads to the hypothesis that an initial interaction of Con-3 and Con-7 with the plasma membrane GnRH-R locally increased the concentration of mitoxantrone, which entered the cell after its release from the conjugates. Mitoxantrone could cross the plasma membrane because it is a lipophilic molecule [53,54]. It is less likely that the endocytosis of the GnRH conjugates/GnRH-R complex might drive the conjugated mitoxantrone into the cell, where it exerts cytotoxic actions after its release. If this were the case, the intracellular fluorescence intensity of mitoxantrone from Con-3 or Con-7 would be smaller than that of its free unconjugated form (control). According to receptor theory, only a small amount of the added ligand binds to its receptor [55]. In contrast, the fluorescent intensities and thus the intracellular concentrations of mitoxantrone in the free and the conjugated forms were similar. Moreover, the human GnRH-R internalizes slowly due to the lack of a cytoplasmic carboxyl-terminal tail [23,56,57,58,59].

Our confocal microscopy experiments revealed that cisplatin, a potent inhibitor of thioredoxin, abolished the accumulation of the conjugated mitoxantrone in Con-3 and Con-7 within the SKOV-3 cells [46,47]. The thioredoxin is an enzyme that catalyzes the reduction in disulfide bonds and it is overexpressed in cancer cells where it is detected both intracellularly and extracellularly [43,44,45,46,60,61,62,63]. These results suggest that the release of mitoxantrone from Con-3 and Con-7 was due to the reduction in their disulfide bonds by the thioredoxin of SKOV-3 cells. This reaction most likely occurred outside the cell because its inhibition by cisplatin resulted in uncleavable conjugates, which are less lipophilic than the free unconjugated form of mitoxantrone and therefore unable to cross the plasma membrane and accumulate within the SKOV-3 cells. Accordingly, cisplatin did not affect the accumulation of the free unconjugated form of the lipophilic mitoxantrone. Similarly, the intracellular accumulation of anthraquinone conjugated through a disulfide bond to the 85–99 epitope of the myelin basic protein within the Jurkat cells was abolished by cisplatin [64].

Interestingly, Con-3 and Con-7, the leuprolide, Con-P1 and Con-P2, but not mitoxantrone, were potent agonists, stimulating calcium mobilization with high potencies (0.46–2 nM). However, the agonistic properties of Con-P1, Con-P2 and leuprolide did not confer cytotoxicity on these peptides as observed in previous studies. The GnRH agonists, triptorelin, decapeptyl GnRH and goserelin, exerted cytotoxic effects on ovarian or endometrial cancer cells, whereas the growth rate of prostate cancer cells was decreased by both leuprolide and triptorelin [22,25,30,31,65,66,67]. However, contradictory results were observed in different studies. For example, leuprolide decreased the cell growth of endometrial carcinomas, whereas triptorelin did not induce apoptosis in EFO-21 and EFO-27 ovarian cancer cell lines [68,69]. Moreover, similar to our results, leuprolide and the GnRH agonist, buserelin, were not cytotoxic in SKOV-3 cells and endometrial cancer cells, respectively [70,71]. It is possible that the differential cytotoxic effects of various GnRH analogs on different cancer cells could be attributed to the different cellular environments and/or the diverse signaling pathways stimulated by divergent conformational states of GnRH-R, which are stabilized by various GnRH agonists [57,72,73,74]. It is also possible that different receptor densities in various cells might affect the biological responses of even the same agonist [74]. Chen et al. have shown that low doses of GnRH analogs enhanced cell invasion in OVCAR-3 ovarian cancer cells but were ineffective in SKOV-3 cells in which the expression levels of GnRH-R were lower than those in OVCAR-3 cells [23]. It is also possible that the introduction of sulfhydryl groups in the leuprolide analogs, Con-P1 and Con-P2, that are required to link the peptides with mitoxantrone (thus creating the Con-3 and Con-7 analogs), might alter their functional properties. 

In conclusion, the GnRH-R in ovarian cancer cells likely serves as a docking place for our GnRH-mitoxantrone conjugates, Con-3 and Con-7, which concentrates the mitoxantrone near the cell surface and exposes it to the extracellular thioredoxin system for release. Subsequently, the lipophilic released form of mitoxantrone is likely to passively enter the cancer cell. In contrast, the GnRH-mitoxantrone conjugates are less lipophilic due to the presence of the peptide moieties, thus being unable to cross the plasma membrane of cells. Thus, theoretically, our GnRH-mitoxantrone conjugates are expected to be less toxic than the free unconjugated mitoxantrone, being less active in non-expressing GnRH-R cells. Our GnRH-mitoxantrone conjugates, Con-3 and Con-7, seem to be promising cytotoxic compounds, acting as “prodrugs” in which mitoxantrone is delivered in a GnRH-R-specific manner. These analogs could be used to selectively target ovarian cancer cells which were not investigated and this could be investigated using xenograft models of ovarian cancer.

## 4. Materials and Methods

### 4.1. Calcium-Based Kinetic Assays of GnRH-R Agonism

HEK293 cells (Cat. No. CRL-1573) obtained from the American Type Culture Collection (ATCC, Mannassas, VA, USA) were transiently transfected with a human GnRH-R plasmid (Gene Bank Accession No. AY39201) obtained from the UMR cDNA Resource Center (Rolla, MO, USA). Transfections were performed using Lipofectamine Plus Reagent (Cat No. A12621; Thermo Fisher Sci., Waltham, MA, USA). Two days post-transfection, the HEK293 cells were harvested via trypsinization to obtain single-cell suspensions that were plated at 80% density on rat tail collagen-coated 96-well Costar 3904 plates for loading with the ratiometric Ca^2+^ indicator fura-2. Measurements of the intracellular [Ca^2+^] were performed using a Flexstation 3 microplate reader (Molecular Devices, Sunnyvale, CA, USA). Briefly, test solutions containing GnRH-R agonists were administered to individual wells of a 96-well plate using the automated pipetting feature of the Flexstation under computer control. Spectrofluorimetry was performed using excitation light delivered at 335/9 and 375/9 nm (center/bandpass wavelengths) in combination with a 455 nm dichroic mirror. Emitted light was detected at 505/15 nm, and the ratio of emission light intensity due to excitation at 335/9 and 375/9 nm was calculated. For data presentation, the y-axis values at individual time points correspond to the mean ± S.D. percent change in the fura-2 ratio value (Δ340/380) for N = 12 wells after baseline subtraction, so that a value of 0.5 indicates a 50% increase in the ratio. The repeatability of these findings was confirmed by performing all experiments a minimum of three times.

### 4.2. Cell Proliferation Assays 

SKOV-3 cells (kindly provided by Prof. Dimitrios Mavroudis, University of Crete) were seeded into 96-well plates at a density of 6000 cells/well, in triplicates, and incubated overnight in Dulbecco’s modified Eagle’s medium/F12 (DMEM/F12) (1:1), containing 3.15 g/L glucose and 10% bovine calf serum (BCS, HyClone), at 37 °C and 5% CO_2_. At the end of the incubation, the culture medium was replaced with a fresh one containing 3.15 g/L glucose and 2% BCS, with or without increasing concentrations of GnRH analogs or mitoxantrone (0.1 nM–10 µM), and the cells were further incubated at 37 °C and 5% CO_2_ for 2, 3 and 4 days. Subsequently, (3-(4,5-dimethylthiazol-2-yl)-2,5- diphenyl tetrazolium bromide) (MTT) was added at a final concentration of 0.5 mg/mL, and the mixtures were incubated for 4 h at room temperature in the dark, allowing the viable cells to reduce the MTT to formazan crystals. The supernatants were removed, and the purple formazan crystals were dissolved in isopropanol containing 4% HCl (100 μL/well) by mixing thoroughly for 30 min in the dark, before the quantification of the absorbance at 594 nm using a Dynatech MicroElisa reader (Chantilly, VA, USA). The percentage of the proliferation rate (% proliferation rate) treated with a GnRH analog was determined based on the following equation: % proliferation rate = (absorbance of cells treated with a GnRH analog/absorbance of untreated cells) × 100. The % viability of the untreated cells (control) was defined as 100%. The % viabilities were analyzed by nonlinear regression analysis, using Prism 8.0 (GraphPad Software, Prism 8.0, San Diego, CA, USA). The half-maximal inhibitory concentration (IC_50_) of GnRH analogs or mitoxantrone, which is the concentration that decreases cell proliferation by 50%, was obtained by fitting the data using nonlinear regression analysis to a sigmoidal dose–response curve. 

### 4.3. Cell Apoptosis Assays 

#### 4.3.1. Annexin V Assay

SKOV-3 cells were seeded into 12-well plates at a density of 50,000 cells/well and incubated overnight in DMEM/F12 (1:1) containing 3.15 g/L glucose and 10% BCS, at 37 °C and 5% CO_2_. At the end of the incubation, the culture medium was replaced with a fresh one containing 3.15 g/L glucose and 2% BCS, with or without GnRH analogs or mitoxantrone at a concentration of 1 μΜ, and the cells were further incubated at 37 °C and 5% CO_2_ for 2, 3 and 4 days. At the end of the incubation, the cells were harvested, and, along with their supernatants (that contain some containing “floating” apoptotic cells that have detached from the monolayer), were centrifuged at 300 g for 5 min at room temperature. The pellets were resuspended in phosphate-buffered saline (PBS) (4.3 mM Na_2_HPO_4_7H_2_O, 1.4 mM KH2PO4, 137 mM NaCl and 2.7 mM KCl, pH 7.3–7.4, at 37 °C) and centrifuged again at 300 g for 5 min at room temperature. The cells were resuspended at 80,000–100,000 cells/mL in the binding buffer (supplied by Biotium, (CF488A Annexin V and PI Apoptosis Detection kit, Biotium, Cat. No. 30061)), aliquoted into flow cytometry tubes at 100 μL/tube and stained with propidium iodide (PI) (Ex/Em: 530/622 nm), (1–2 μL/aliquot) and CF488A-conjugated annexin V (Ex/Em: 490/515 nm), (5 μL/aliquot), (CF488A Annexin V and PI Apoptosis Detection kit, Biotinum, Cat. No. 30061). After incubation at room temperature for 30 min in the dark, 400 μL binding buffer was added to each aliquot and the cells were analyzed using flow cytometry on a BD FACS Calibur flow cytometer (excitation 488 nm), in the FITC channel (filter 530 nm) for detection of the Annexin V-positive cells) and in the PI channel (filter 616 nm) for the detection of the PI-positive cells. The data were analyzed using the using the FlowJo™ v10 software. The flow cytometry analysis of apoptosis was presented as a dot plot scattergram showing the viable, necrotic and apoptotic cells or as a graph showing the time-course quantification of the percentage of apoptotic cells. In the dot plots, the right lower quadrant Q1 (annexin positive and PI negative) represents early apoptotic cells (cells that retain membrane integrity), the right upper quadrant Q2 (both annexin and PI positive) represents late apoptotic cells (cells with compromised membranes), the left upper quadrant Q3 (PI positive and annexin negative) represents necrotic and late apoptotic cells and the left lower quadrant Q4 (both annexin and PI negative) represents live, non-apoptotic, cells [75,76]. The percentage of apoptotic cells was determined by dividing the number of apoptotic cells by the total number of cells and then multiplying by 100. 

#### 4.3.2. TMRE-Based Detection of Mitochondrial Membrane Potential

The reduced mitochondrial membrane potential (ΔΨm) that accompanies apoptosis was detected using the fluorescent dye, tetramethylrhodamine ethyl ester (TMRE) (Ex/Em: 530/580 nm), (Cayman, TMRE Mitochondrial Membrane Potential Assay Kit, Item No. 701310). SKOV-3 cells were grown on 60 mm tissue culture dishes to achieve ca. 60% confluence. Monolayers of these cells were treated for a total of 72 h using RPMI 1640 containing 1 μM each of Con-3, Con-7 or mitoxantrone. At the end of each consecutive 24 h treatment period, the test solutions were removed and replaced with new media containing fresh test compounds. After 72 h TMRE was added to each dish to a final concentration of 5 μM. After a 30 min exposure to TMRE, the media was removed so that the cell monolayers could be dispersed using 0.25% trypsin-EDTA. The resultant cell suspensions were subjected to centrifugation and repeated resuspension in a standard extracellular salt solution (SES). Aliquots of these suspensions were then added to individual wells of a black 96-well clear bottom plate. The TMRE fluorescence intensities monitored from the individual wells were measured using a Flexstation 3 microplate reader (Molecular Devices, Sunnyvale, CA, USA).

### 4.4. Confocal Microscopy

SKOV-3 cells were seeded into 24-well plates onto glass slides at a density of 30.000 cells/well and incubated overnight in DMEM/F12 (1:1) containing 3.15 g/L glucose and 10% BCS, at 37 °C and 5% CO_2_. At the end of the incubation, the culture medium was replaced with a fresh one containing 3.15 g/L glucose and 2% BCS, with or without GnRH analogs or mitoxantrone at a concentration of 1μΜ, and the cells were further incubated at 37 °C and 5% CO_2_ for 2, 3 and 4 days. For cells treated with the GnRH analogs, a group of them was pretreated with cisplatin (30 μg/mL) for 1 h at 37 °C and 5% CO_2_ before adding the GnRH analogs. At the end of the incubation, the cells were washed with PBS and fixed with 4% paraformaldehyde (PFA). The cell nuclei were stained with the DNA-staining Hoechst 33342 dye (neoFroxx, Cat. No. 2289GR001) at a 1:10,000 dilution [42]. Cell-containing coverslips were mounted onto glass slides using one drop of 80% glycerol. Confocal fluorescent images were obtained using a Leica SP8 microscope and a 63X magnification lens. Sequential excitation at 350 nm (for the Hoechst dye) and 638 nm (for the mitoxantrone which fluoresces with excitation maxima at 610 and 660 nm and emission maximum at 685 nm) was provided by the appropriate lasers [41]. The emission filters of 461 nm and 650 nm–700 nm were used for collecting blue (Hoechst dye) and red (mitoxantrone) signals in separate channels. 

### 4.5. Quantification of the Fluorescence Intensity

The quantitative analysis of fluorescence intensity of compound-treated and untreated SKOV-3 cells was performed using the Image J software. Images of cells exported from the LAS X acquisition software of the Leica SP8 microscope were analyzed and mean fluorescence intensities were determined. 

## Figures and Tables

**Figure 6:**
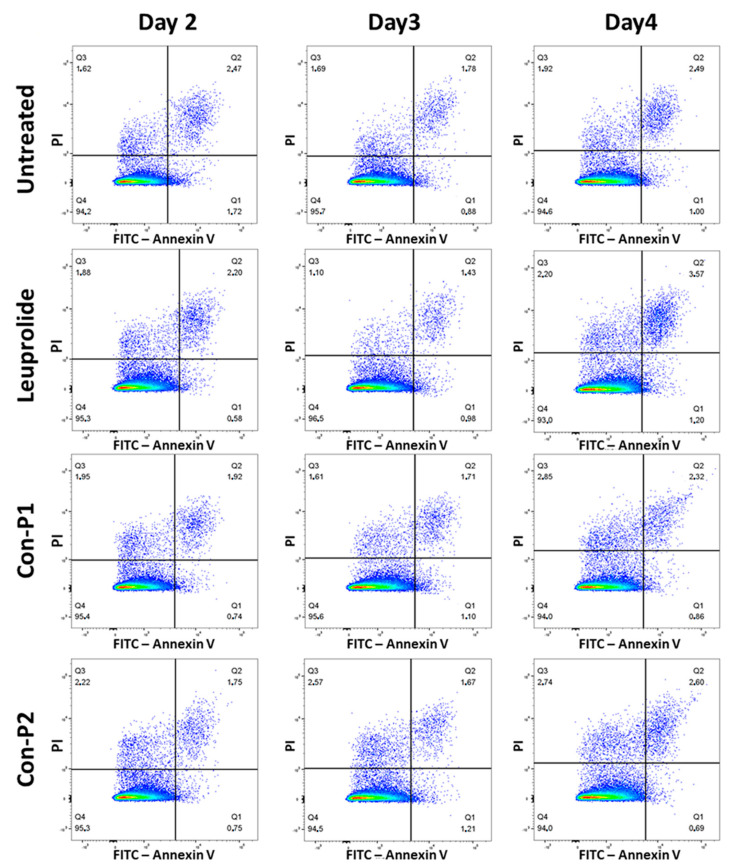
Determination of viability, apoptosis or necrosis of untreated SKOV-3 cells or treated with 1 μM leuprolide, Con-P1, or Con-P2 for 2, 3, and 4 days at 37 °C. At the end of the incubation the cells were further incubated in binding buffer supplied by Biotium, (CF488A Apoptosis Detection kit) and analyzed on a flow cytometer (excitation 488 nm), in the FITC channel (filter 530 nm) for the detection of the Annexin V-positive cellsand in the PI channel (filter 616 nm) for the detection of the PI-positive cells. The figures are from a representative experiment performed two or three times with similar results. A higher resolution of the images in Figure 6 is provided in the Supplementary Material (Figures S10–S21).

**Table 1:** The determination of the percentage of apoptotic cells (Q1 and Q2) using flow cytometry. The data from three experiments were statistically analyzed by a Day-Group Interaction analysis using repeated measures ANOVA. The asterisks indicate that the percentage of apoptotic cells treated with the Con-3, Con-7 or mitoxantrone is significantly higher (*p* < 0.01; post hoc Bonferroni analysis, ANOVA) than that of untreated cells (control).

Apoptotic Cells (%)	Control		Con-3		Con-7		Mitoxantrone	
	Mean	SEM	Mean	SEM	Mean	SEM	Mean	SEM
Day2	2.12	0.67	13.68 *	1.87	13.81 *	1.58	14.26 *	0.62
Day3	1.96	0.35	35.00 *	3.50	33.53 *	5.91	33.60 *	3.00
Day4	3.69	0.70	49.50 *	2.04	51.17 *	4.22	50.10 *	2.15

**Table 2:** Quantification of the fluorescence intensity of confocal microscopy images after 6 h incubation of cells with the compounds (Figure 9, Figure 10 and Figure 11). The quantification of fluorescence intensity was accomplished using the Image J software. Specifically, the fluorescence intensity of 3 or more treated cells per compound or untreated cells (control) was quantified and the mean value ± SEM values were determined. Control values correspond to the background and autofluorescence recorded in the mitoxantrone channel (638 nm). The fluorescence intensities of Con-3, Con-7 and mitoxantrone are not statistically different, as determined by *t*-test statistical analysis. Preincubation with cisplatin significantly decreased the fluorescence intensities (*p* < 0.05) of Con-3 and Con-7 but not that of mitoxantrone.

	Fluorescence Intensity	SEM
Con-7	9738	398.4
Con-7 + cisplatin	3244	236.7
Con-3	9015	243.6
Con-3 + cisplatin	2978	161.7
Mitoxantrone	9078	396.1
Mitoxantrone + cisplatin	9021	16.2
Control	583	23.1

## Data Availability

Data are contained within the article.

## Supplementary Materials

The following supporting information can be downloaded at https://www.mdpi.com/article/10.3390/molecules29174127/s1. The following supporting information contains Figures S1–S21. Figure S1: SKOV-3 cells treated with 1 μM Con-3, for 2 days; Figure S2: SKOV-3 cells treated with 1 μM Con-7, for 2 days; Figure S3: SKOV-3 cells treated with 1 μM mitoxantrone, for 2 days; Figure S4: SKOV-3 cells treated with 1 μM Con-3, for 3 days; Figure S5: SKOV-3 cells treated with 1 μM Con-7, for 3 days; Figure S6: SKOV-3 cells treated with 1 μM mitoxantrone, for 3 days; Figure S7: SKOV-3 cells treated with 1 μM Con-3, for 4 days; Figure S8: SKOV-3 cells treated with 1 μM Con-7, for 4 days; Figure S9: SKOV-3 cells treated with 1 μM mitoxantrone, for 4 days; Figure S10: Untreated SKOV-3 cells at day 2; Figure S11: SKOV-3 cells treated with 1 μM leuprolide, for 2 days; Figure S12: SKOV-3 cells treated with 1 μM Con-P1, for 2 days; Figure S13: SKOV-3 cells treated with 1 μM Con-P2, for 2 days; Figure S14: Untreated SKOV-3 cells at day 3; Figure S15: SKOV-3 cells treated with 1 μM leuprolide, for 3 days; Figure S16: SKOV-3 cells treated with 1 μM Con-P1, for 3 days; Figure S17: SKOV-3 cells treated with 1 μM Con-P2, for 3 days; Figure S18: Untreated SKOV-3 cells at day 4; Figure S19: SKOV-3 cells treated with 1 μM leuprolide, for 4 days; Figure S20: SKOV-3 cells treated with 1 μM Con-P1, for 4 days; Figure S21: SKOV-3 cells treated with 1 μM Con-P2, for 4 days
