# Supplementary material for: Cytotoxic Activity of Novel GnRH Analogs Conjugated with Mitoxantrone in Ovarian Cancer Cells
Source: Molecules. 2024 Aug 30;29(17):4127. doi: 10.3390/molecules29174127 (PMC11397358; doi:10.3390/molecules29174127)
Supplement: Supplementary file 1 [file molecules-29-04127-s001.zip › molecules-3014265-supplementary.pdf]

# Supplementary Information

# Cytotoxic activity of novel GnRH analogs conjugated with mitoxantrone in ovarian cancer cells

**Christos Markatos[1#], Georgia Biniari[2#], Oleg G. Chepurny[3], Vlasios Karageorgos[1], Nikos Tsakalakis[1], Georgios Komontachakis[1], Zacharenia Vlata[4], Maria Venihaki[5], George G. Holz[6], Theodore Tselios[2*], George Liapakis[1*]**

[1] Department of Pharmacology, School of Medicine, University of Crete, Heraklion, Greece, xristosmarkatos@gmail.com (C.M.), bkarageorgos@hotmail.com (V.K.), chem2763@edu.chemistry.uoc.gr (N.T.), chem2785@edu.chemistry.uoc.gr (G.K.), liapakig@uoc.gr (G.L.)
[2] Department of Chemistry, University of Patras, 26504 Rion, Greece, georgiabiniari96@gmail.com (G.B.), , ttselios@upatras.gr (T.T.)
[3] Department of Medicine, State University of New York (SUNY), Upstate Medical University, Syracuse, New York 13210, ChepurnO@upstate.edu (O.G.C.)
[4] Flow Cytometry Facility, Institute of Molecular Biology and Biotechnology of the Foundation for Research and Technology Hellas (IMBB-FORTH), xvlata@imbb.forth.gr
[5] Department of Clinical Chemistry, School of Medicine, University of Crete, 71003 Heraklion, Greece, venycham@uoc.gr (M.V.)
[6] Department of Medicine and Pharmacology, State University of New York (SUNY), Upstate Medical University, Syracuse, New York 13210, HolzG@upstate.edu (G.G.H.)

*Correspondence: liapakig@uoc.gr (G.L.) and ttselios@upatras.gr (T.T.)
#These authors contributed equally to this work.

**Contents:**

**Figure S1.** SKOV-3 cells treated with 1 μM Con-3, for 2 days
**Figure S2.** SKOV-3 cells treated with 1 μM Con-7, for 2 days
**Figure S3.** SKOV-3 cells treated with 1 μM mitoxantrone, for 2 days
**Figure S4.** SKOV-3 cells treated with 1 μM Con-3, for 3 days
**Figure S5.** SKOV-3 cells treated with 1 μM Con-7, for 3 days
**Figure S6.** SKOV-3 cells treated with 1 μM mitoxantrone, for 3 days
**Figure S7.** SKOV-3 cells treated with 1 μM Con-3, for 4 days
**Figure S8.** SKOV-3 cells treated with 1 μM Con-7, for 4 days
**Figure S9.** SKOV-3 cells treated with 1 μM mitoxantrone, for 4 days
**Figure S10.** Untreated SKOV-3 cells at day 2
**Figure S11.** SKOV-3 cells treated with 1 μM leuprolide, for 2 days
**Figure S12.** SKOV-3 cells treated with 1 μM Con-P1, for 2 days
**Figure S13.** SKOV-3 cells treated with 1 μM Con-P2, for 2 days
**Figure S14.** Untreated SKOV-3 cells at day 3
**Figure S15.** SKOV-3 cells treated with 1 μM leuprolide, for 3 days
**Figure S16.** SKOV-3 cells treated with 1 μM Con-P1, for 3 days
**Figure S17.** SKOV-3 cells treated with 1 μM Con-P2, for 3 days
**Figure S18.** Untreated SKOV-3 cells at day 4
**Figure S19.** SKOV-3 cells treated with 1 μM leuprolide, for 4 days
**Figure S20.** SKOV-3 cells treated with 1 μM Con-P1, for 4 days
**Figure S21.** SKOV-3 cells treated with 1 μM Con-P2, for 4 days

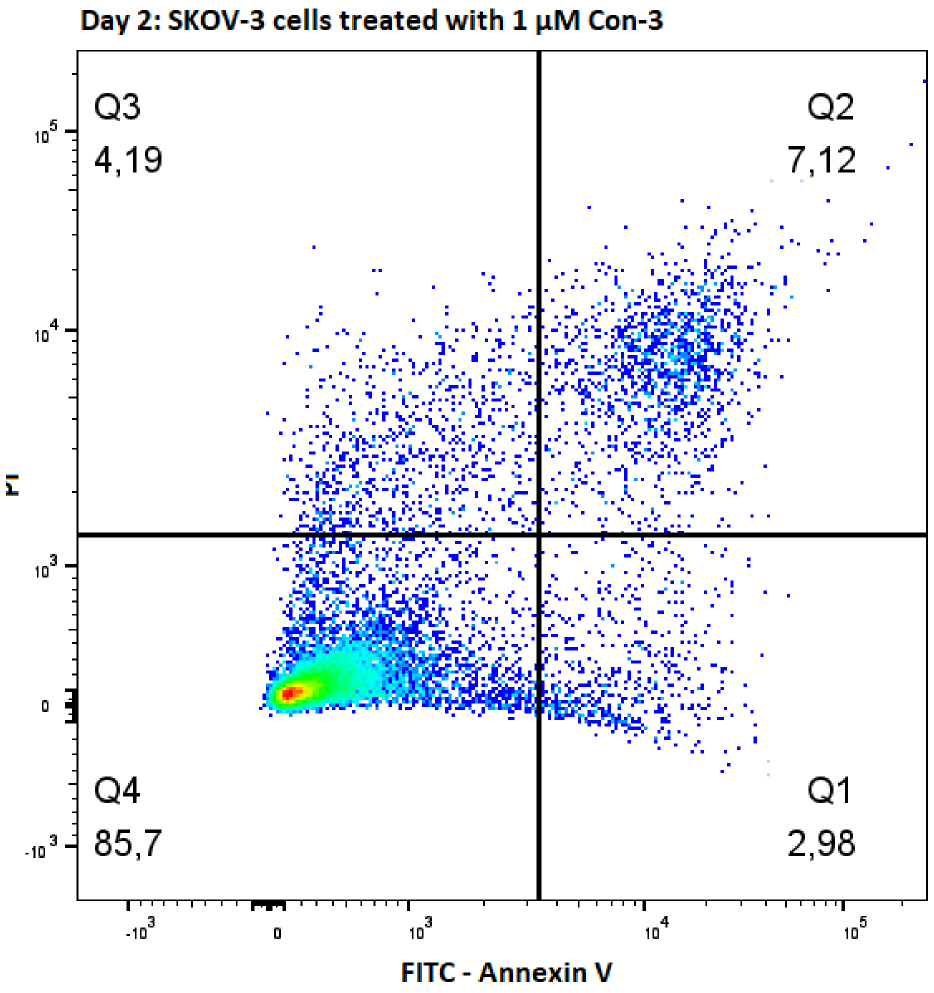


**Figure S1.** Determination of viability, apoptosis, or necrosis of SKOV-3 cells after their treatment with 1 μM Con-3, for 2 days at 37°C. The figure is from a representative experiment performed three times with similar results. The mean ± SEM (Standard Error of the Mean) values of apoptotic cells are shown in Table 1 and Figure 7. Detailed information is provided in the legend of Figure 5.

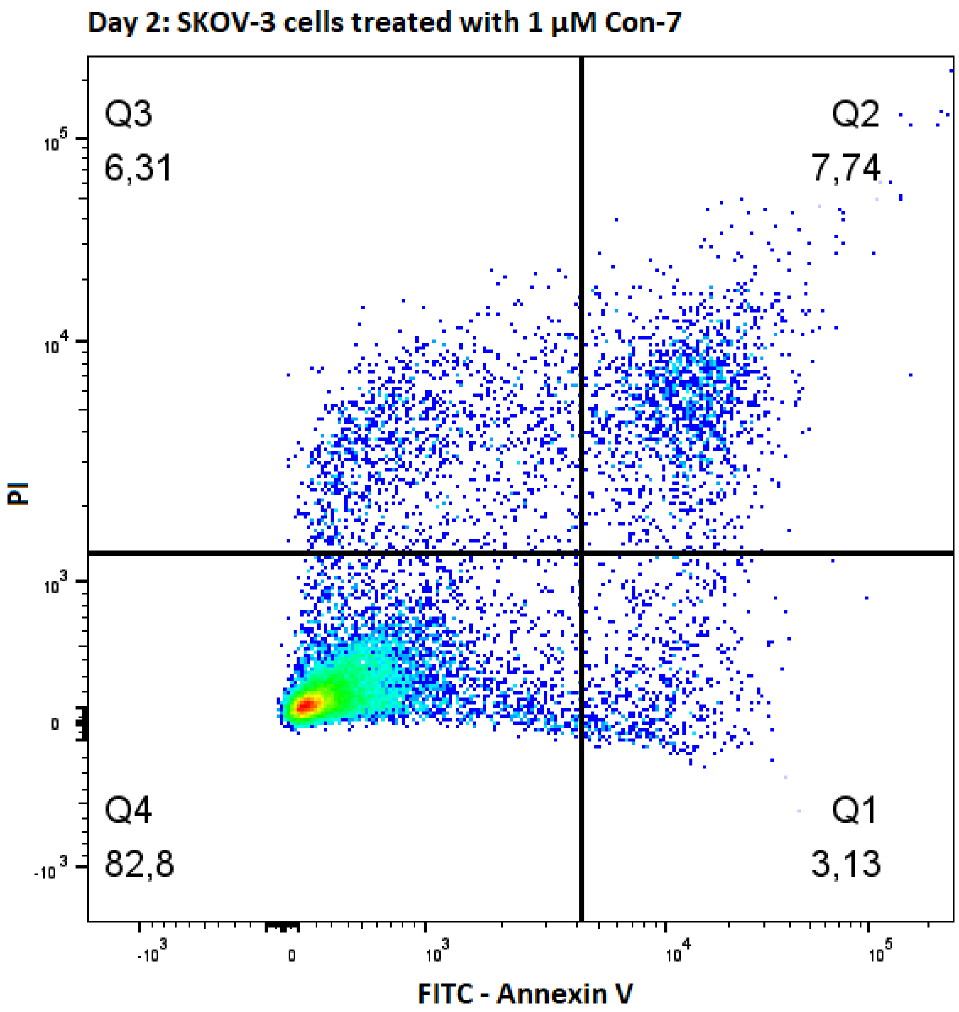


**Figure S2.** Determination of viability, apoptosis, or necrosis of SKOV-3 cells after their treatment with 1 µM Con-7, for 2 days at 37°C. The figure is from a representative experiment performed three times with similar results. The mean ± SEM (Standard Error of the Mean) values of apoptotic cells are shown in Table 1 and Figure 7. Detailed information is provided in the legend of Figure 5.

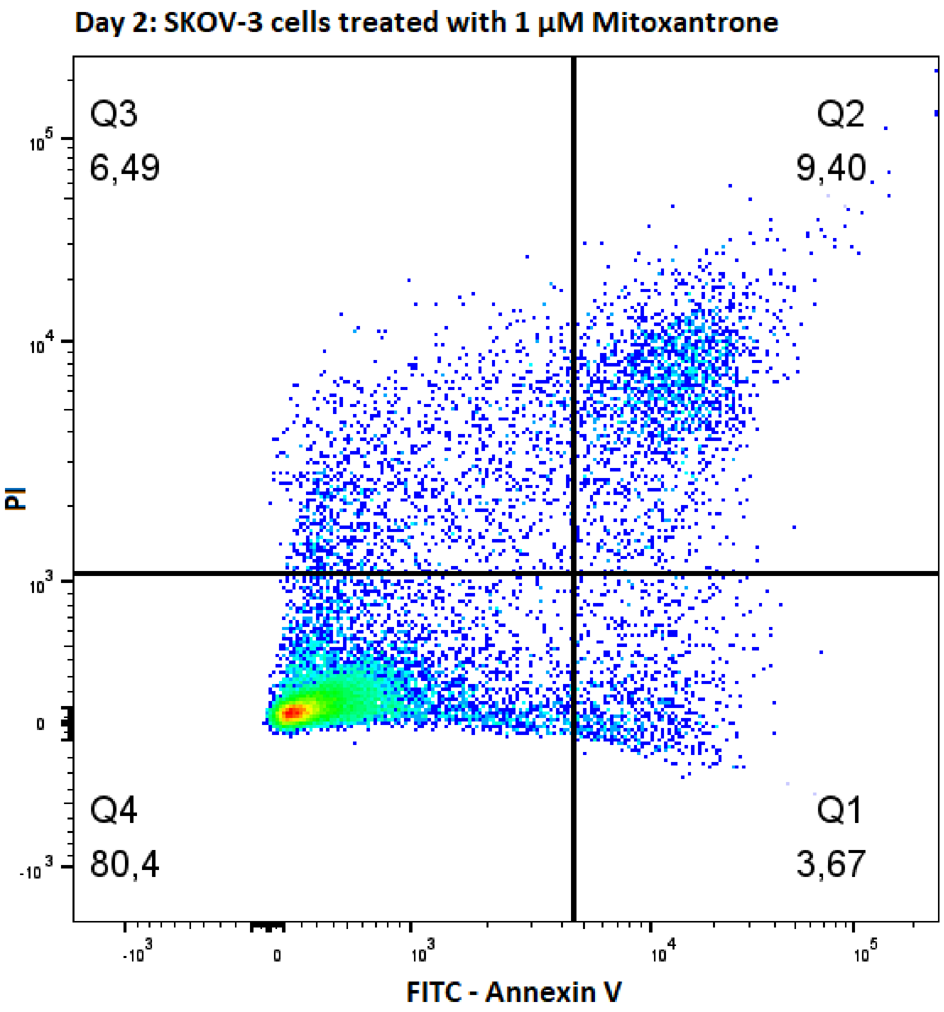


**Figure S3.** Determination of viability, apoptosis, or necrosis of SKOV-3 cells after their treatment with 1 µM mitoxantrone, for 2 days at 37°C. The figure is from a representative experiment performed three times with similar results. The mean ± SEM (Standard Error of the Mean) values of apoptotic cells are shown in Table 1 and Figure 7. Detailed information is provided in the legend of Figure 5.

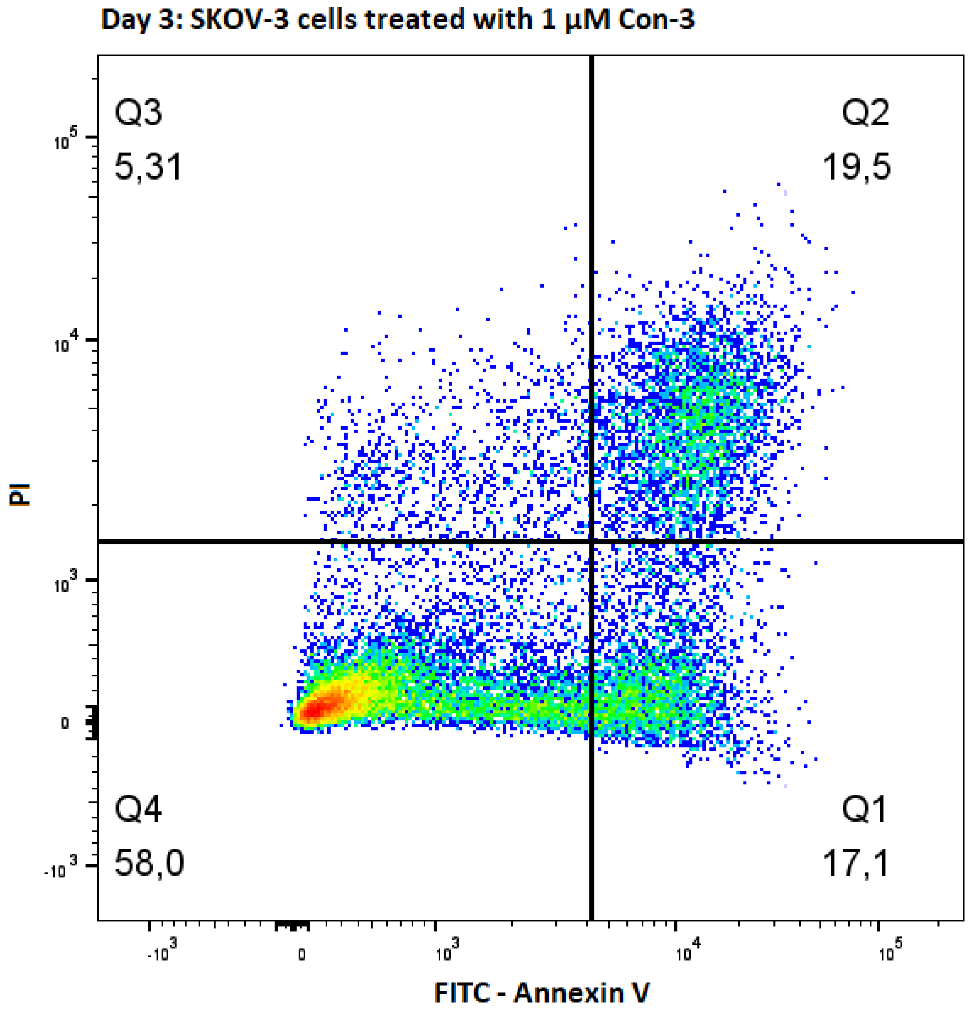


**Figure S4.** Determination of viability, apoptosis, or necrosis of SKOV-3 cells after their treatment with 1 μM Con-3, for 3 days at 37°C. The figure is from a representative experiment performed three times with similar results. The mean ± SEM (Standard Error of the Mean) values of apoptotic cells are shown in Table 1 and Figure 7. Detailed information is provided in the legend of Figure 5.

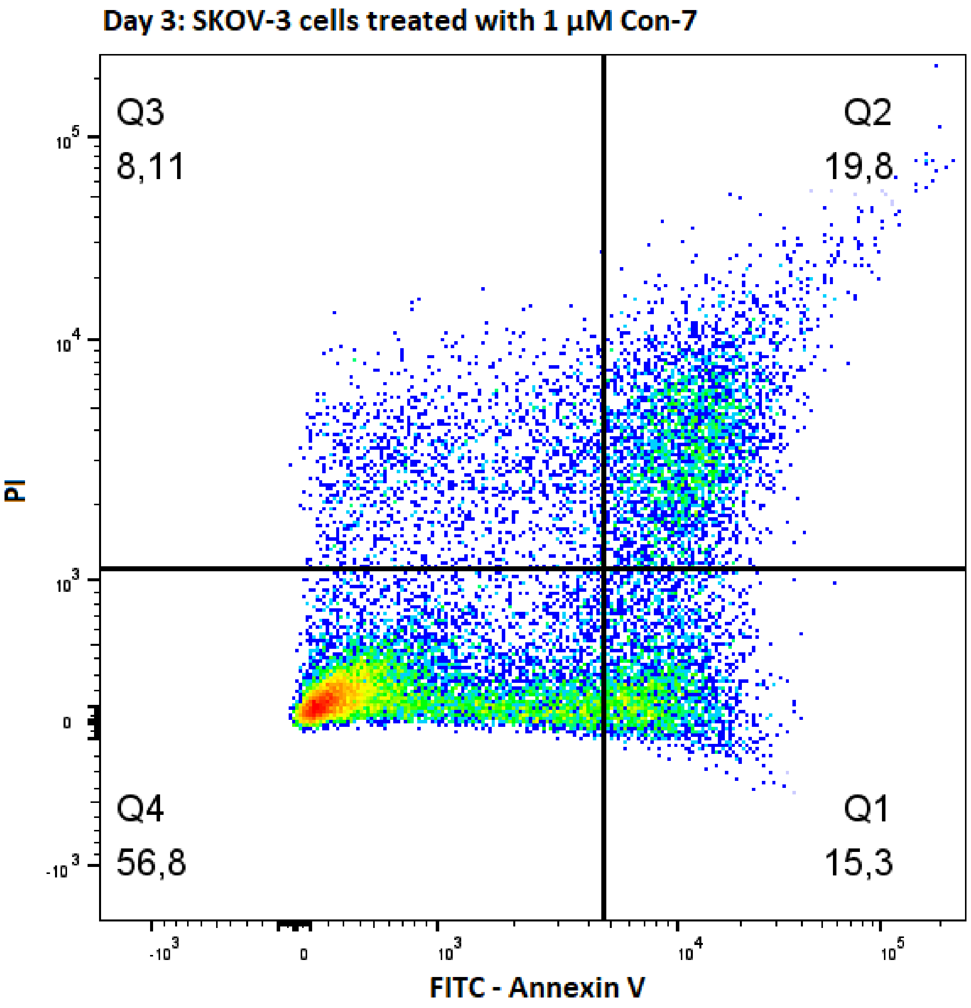


**Figure S5.** Determination of viability, apoptosis, or necrosis of SKOV-3 cells after their treatment with 1 µM Con-7, for 3 days at 37°C. The figure is from a representative experiment performed three times with similar results. The mean ± SEM (Standard Error of the Mean) values of apoptotic cells are shown in Table 1 and Figure 7. Detailed information is provided in the legend of Figure 5.

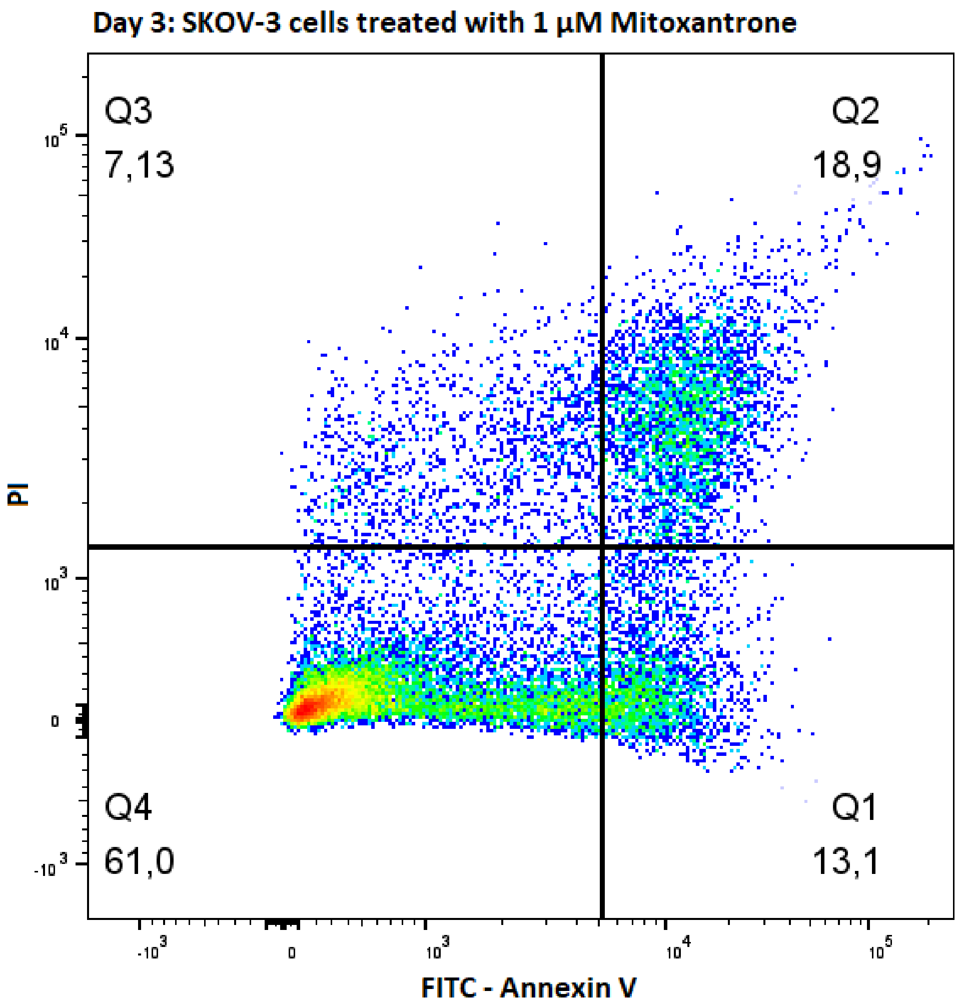


**Figure S6.** Determination of viability, apoptosis, or necrosis of SKOV-3 cells after their treatment with 1 µM mitoxantrone, for 3 days at 37°C. The figure is from a representative experiment performed three times with similar results. The mean ± SEM (Standard Error of the Mean) values of apoptotic cells are shown in Table 1 and Figure 7. Detailed information is provided in the legend of Figure 5.

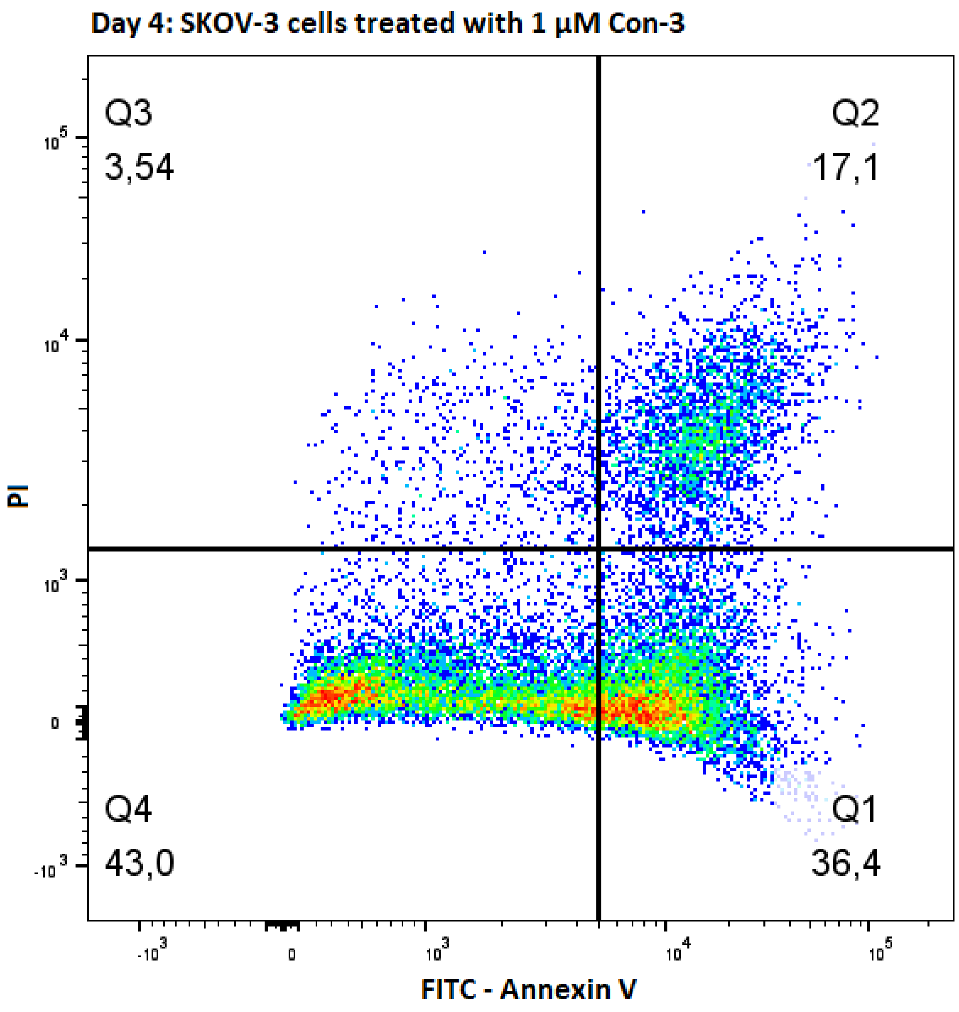


**Figure S7.** Determination of viability, apoptosis, or necrosis of SKOV-3 cells after their treatment with 1 µM Con-3, for 4 days at 37°C. The figure is from a representative experiment performed three times with similar results. The mean ± SEM (Standard Error of the Mean) values of apoptotic cells are shown in Table 1 and Figure 7. Detailed information is provided in the legend of Figure 5.

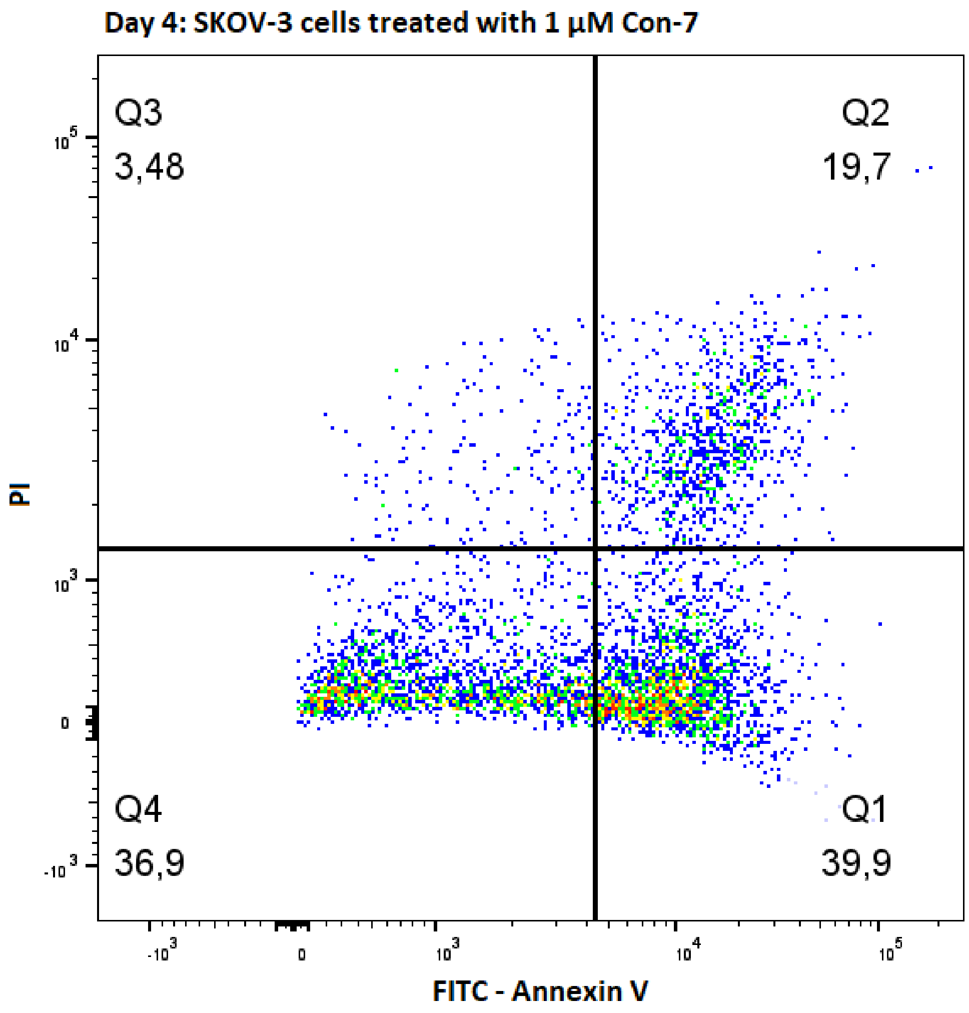


**Figure S8.** Determination of viability, apoptosis, or necrosis of SKOV-3 cells after their treatment with 1 μM Con-7, for 4 days at 37°C. The figure is from a representative experiment performed three times with similar results. The mean ± SEM (Standard Error of the Mean) values of apoptotic cells are shown in Table 1 and Figure 7. Detailed information is provided in the legend of Figure 5.

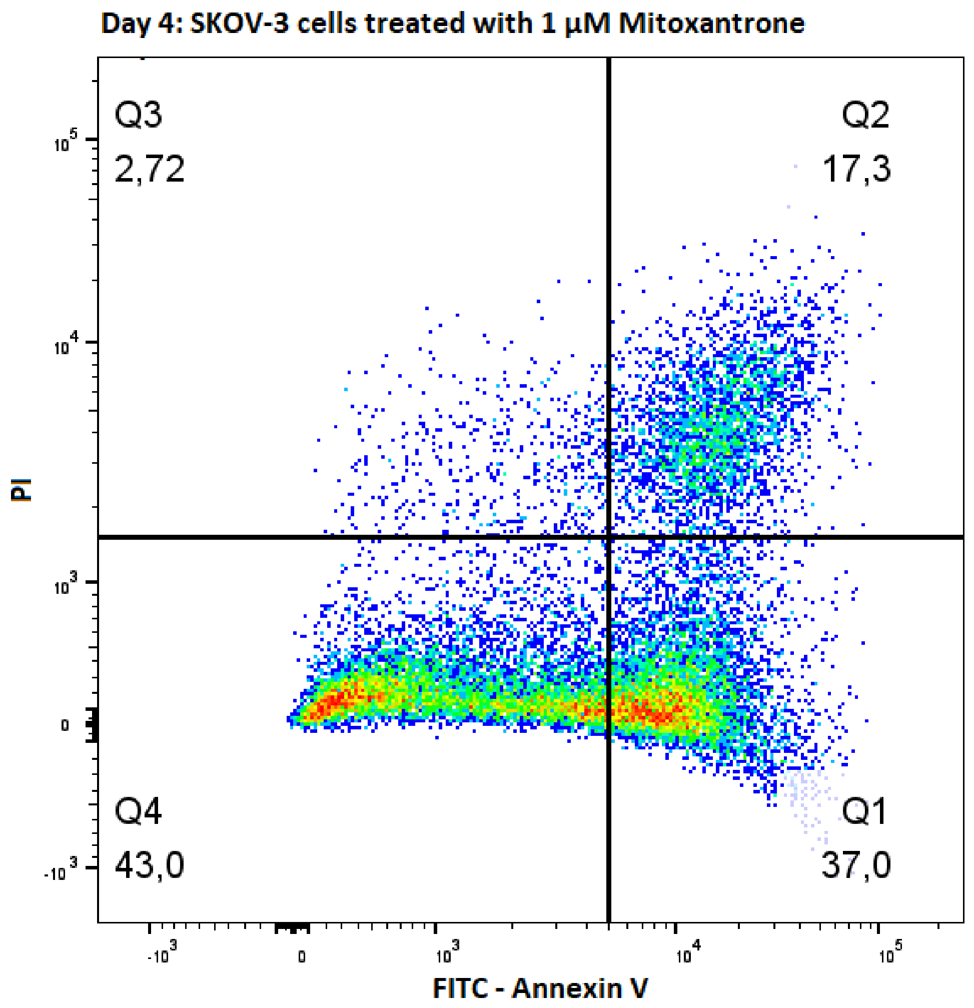


**Figure S9.** Determination of viability, apoptosis, or necrosis of SKOV-3 cells after their treatment with 1 µM mitoxantrone, for 4 days at 37°C. The figure is from a representative experiment performed three times with similar results. The mean ± SEM (Standard Error of the Mean) values of apoptotic cells are shown in Table 1 and Figure 7. Detailed information is provided in the legend of Figure 5.

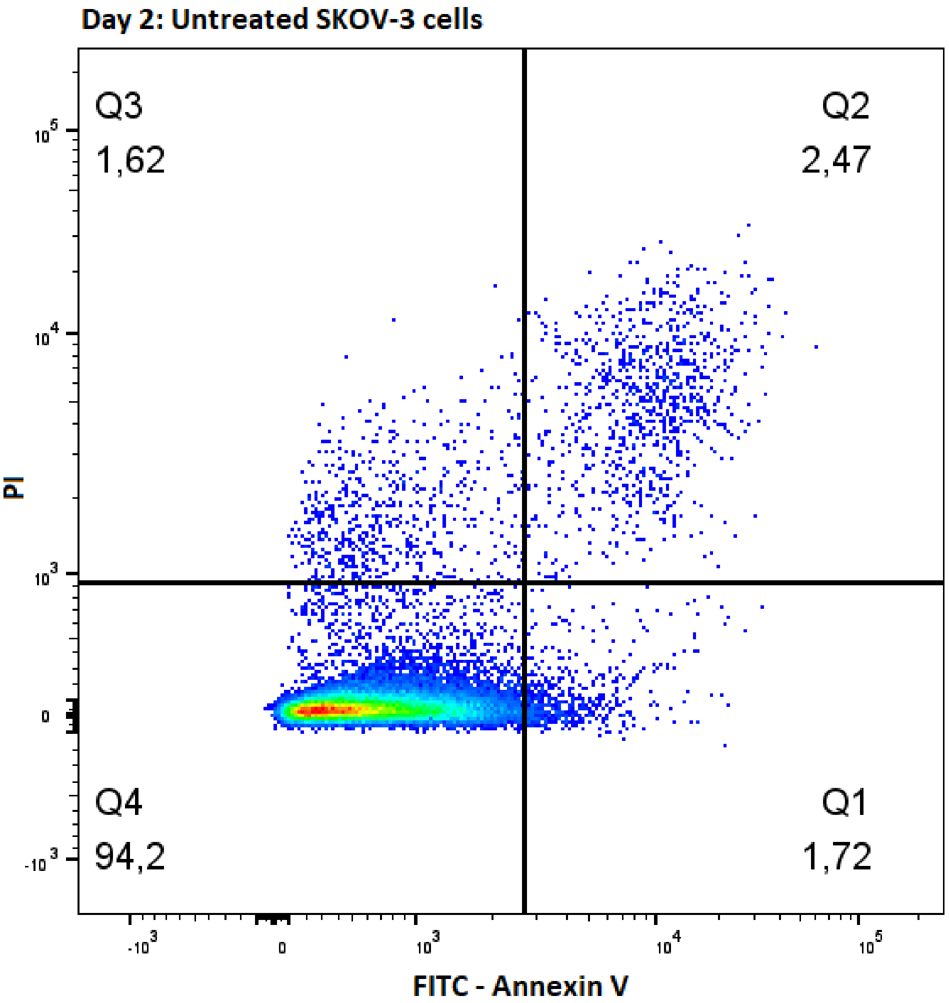


**Figure S10.** Determination of viability, apoptosis, or necrosis of untreated SKOV-3 cells at day 2. The figure is from a representative experiment performed three times with similar results. The mean ± SEM (Standard Error of the Mean) values of apoptotic cells are shown in Table 1 and Figure 7. Detailed information is provided in the legend of Figure 6.

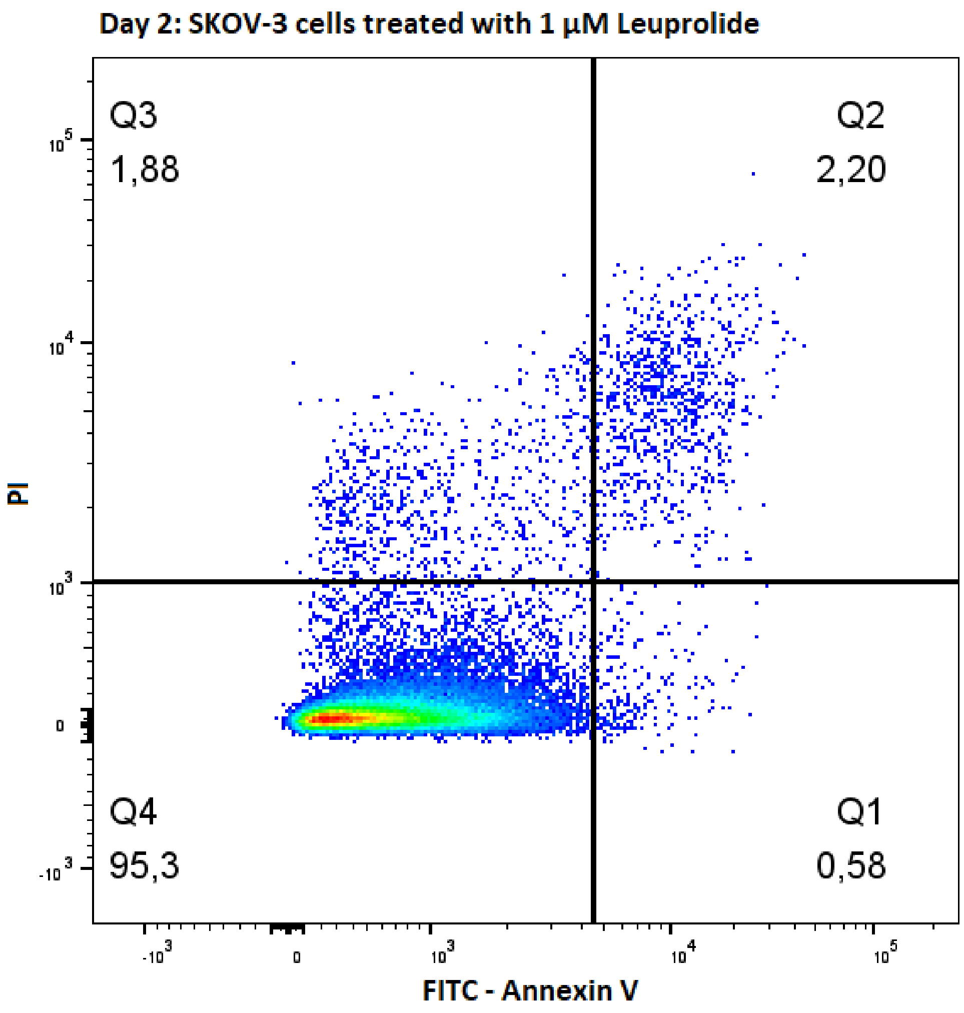


**Figure S11.** Determination of viability, apoptosis, or necrosis of SKOV-3 cells after their treatment with 1 µM leuprolide for 2 days at 37°C. The figure is from a representative experiment performed three times with similar results. The mean ± SEM (Standard Error of the Mean) values of apoptotic cells are shown in Table 1 and Figure 7. Detailed information is provided in the legend of Figure 6.

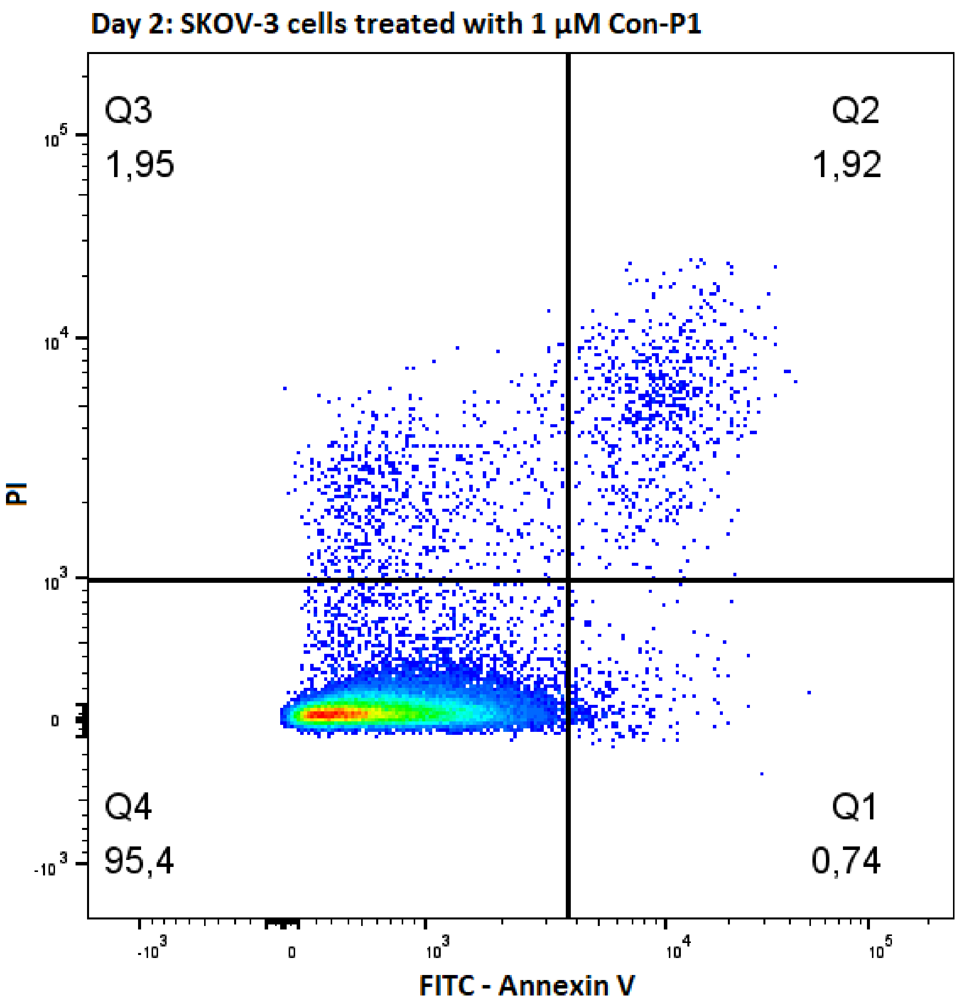


**Figure S12.** Determination of viability, apoptosis, or necrosis of SKOV-3 cells after their treatment with 1 µM Con-P1 for 2 days at 37°C. The figure is from a representative experiment performed three times with similar results. The mean ± SEM (Standard Error of the Mean) values of apoptotic cells are shown in Table 1 and Figure 7. Detailed information is provided in the legend of Figure 6.

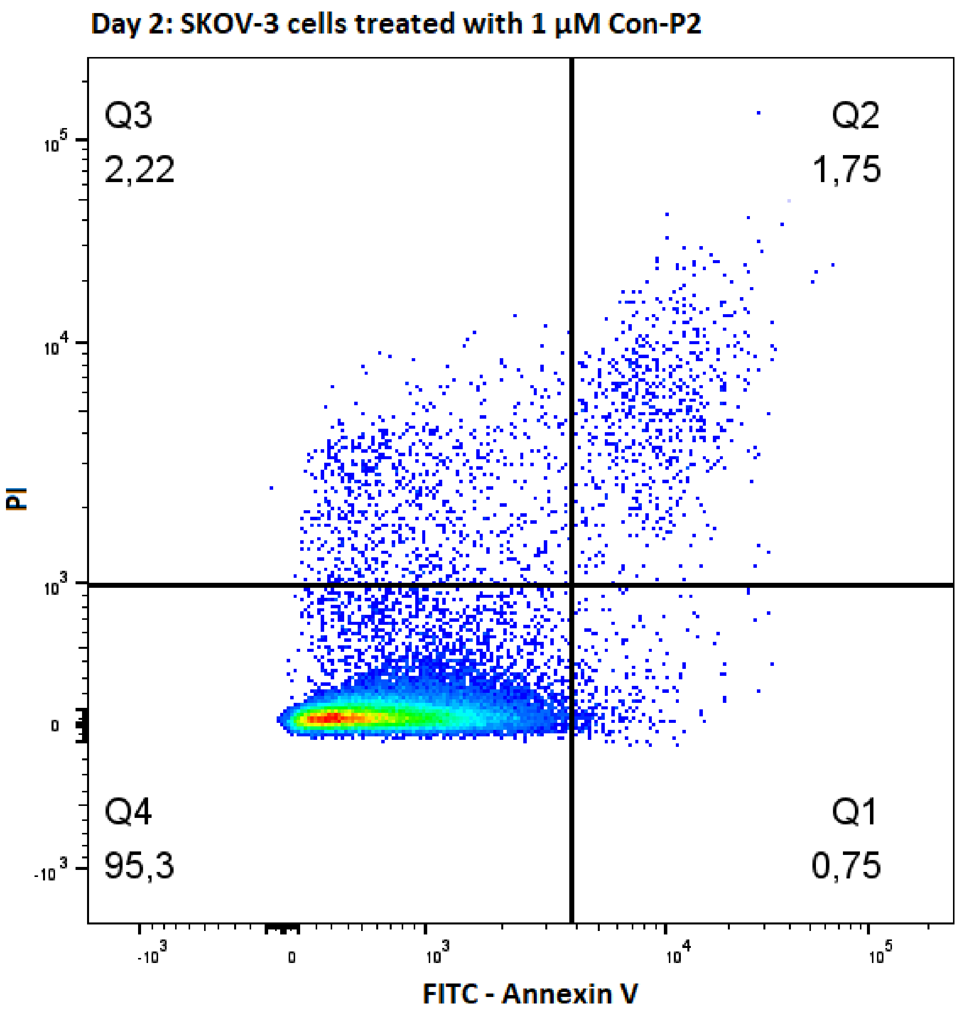


**Figure S13.** Determination of viability, apoptosis, or necrosis of SKOV-3 cells after their treatment with 1 µM Con-P2 for 2 days at 37°C. The figure is from a representative experiment performed three times with similar results. The mean ± SEM (Standard Error of the Mean) values of apoptotic cells are shown in Table 1 and Figure 7. Detailed information is provided in the legend of Figure 6.

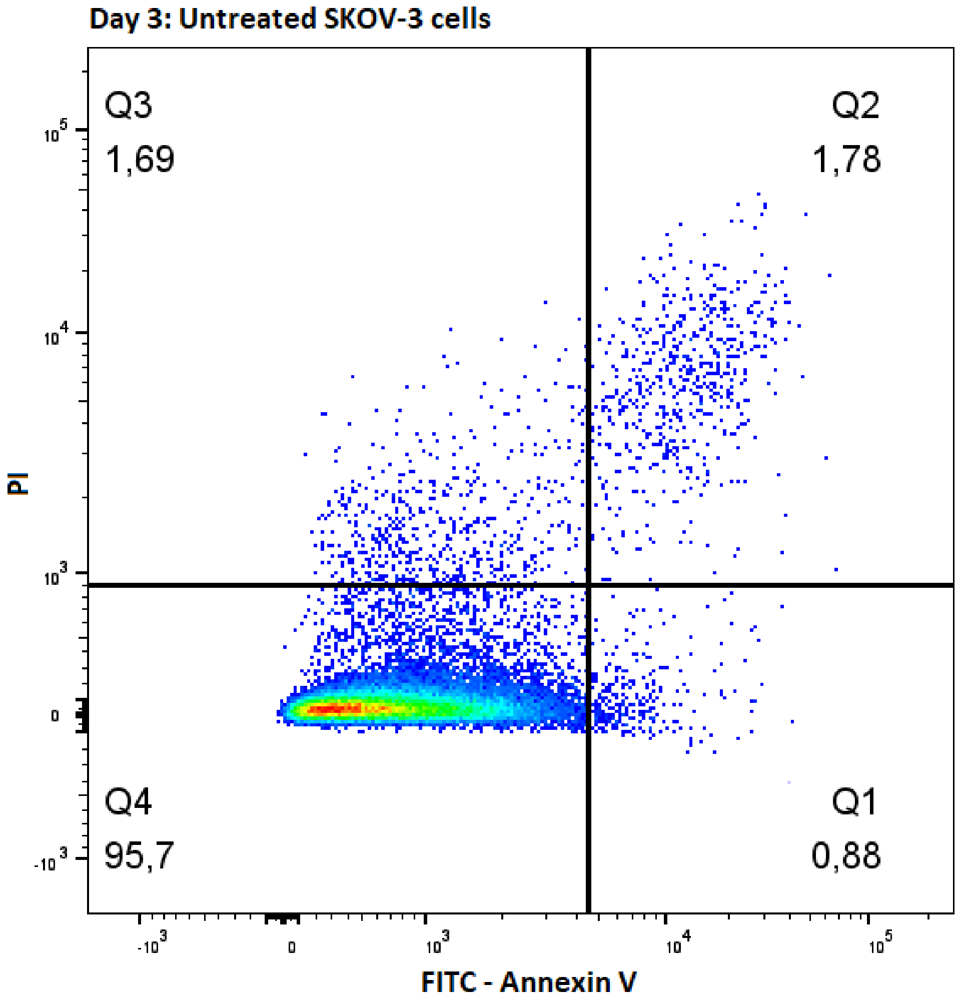


**Figure S14.** Determination of viability, apoptosis, or necrosis of untreated SKOV-3 cells at day 3. The figure is from a representative experiment performed three times with similar results. The mean ± SEM (Standard Error of the Mean) values of apoptotic cells are shown in Table 1 and Figure 7. Detailed information is provided in the legend of Figure 6.

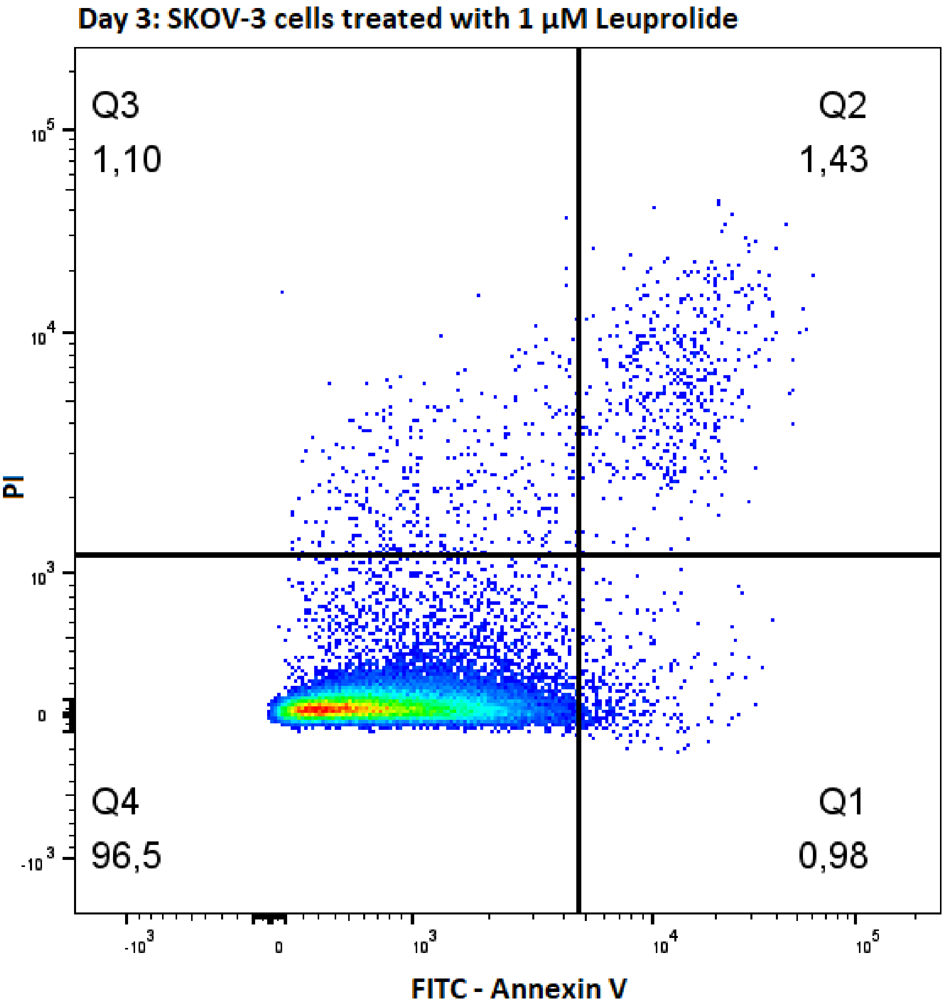


**Figure S15.** Determination of viability, apoptosis, or necrosis of SKOV-3 cells after their treatment with 1 μM leuprolide for 3 days at 37°C. The figure is from a representative experiment performed three times with similar results. The mean ± SEM (Standard Error of the Mean) values of apoptotic cells are shown in Table 1 and Figure 7. Detailed information is provided in the legend of Figure 6.

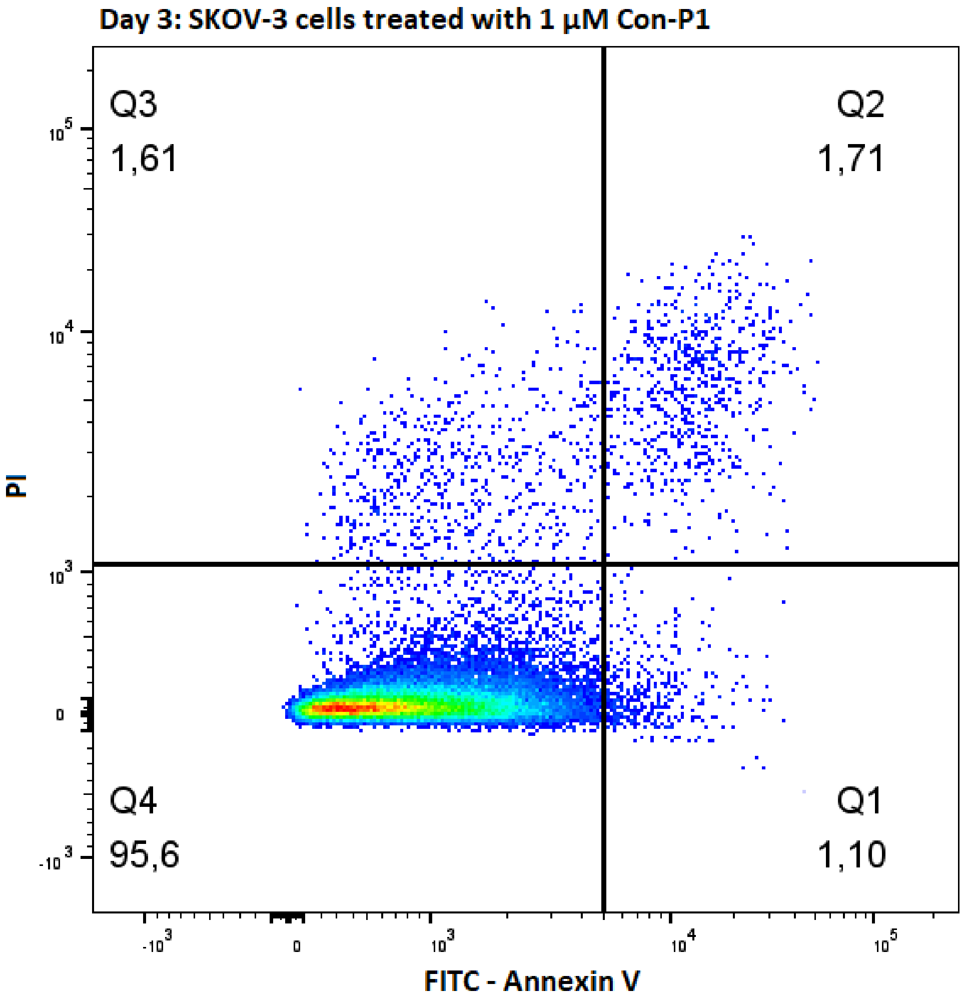


**Figure S16.** Determination of viability, apoptosis, or necrosis of SKOV-3 cells after their treatment with 1 μM Con-P1 for 3 days at 37°C. The figure is from a representative experiment performed three times with similar results. The mean ± SEM (Standard Error of the Mean) values of apoptotic cells are shown in Table 1 and Figure 7. Detailed information is provided in the legend of Figure 6.

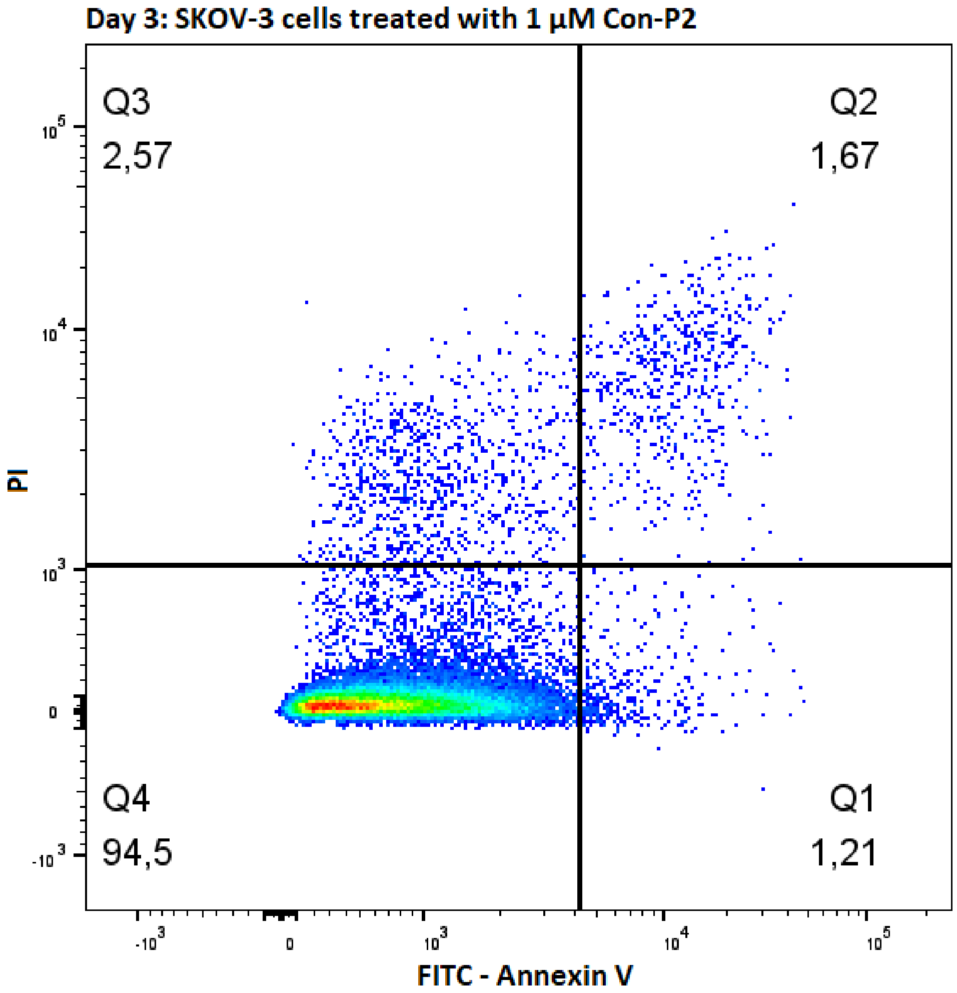


**Figure S17.** Determination of viability, apoptosis, or necrosis of SKOV-3 cells after their treatment with 1 µM Con-P2 for 3 days at 37°C. The figure is from a representative experiment performed three times with similar results. The mean ± SEM (Standard Error of the Mean) values of apoptotic cells are shown in Table 1 and Figure 7. Detailed information is provided in the legend of Figure 6.

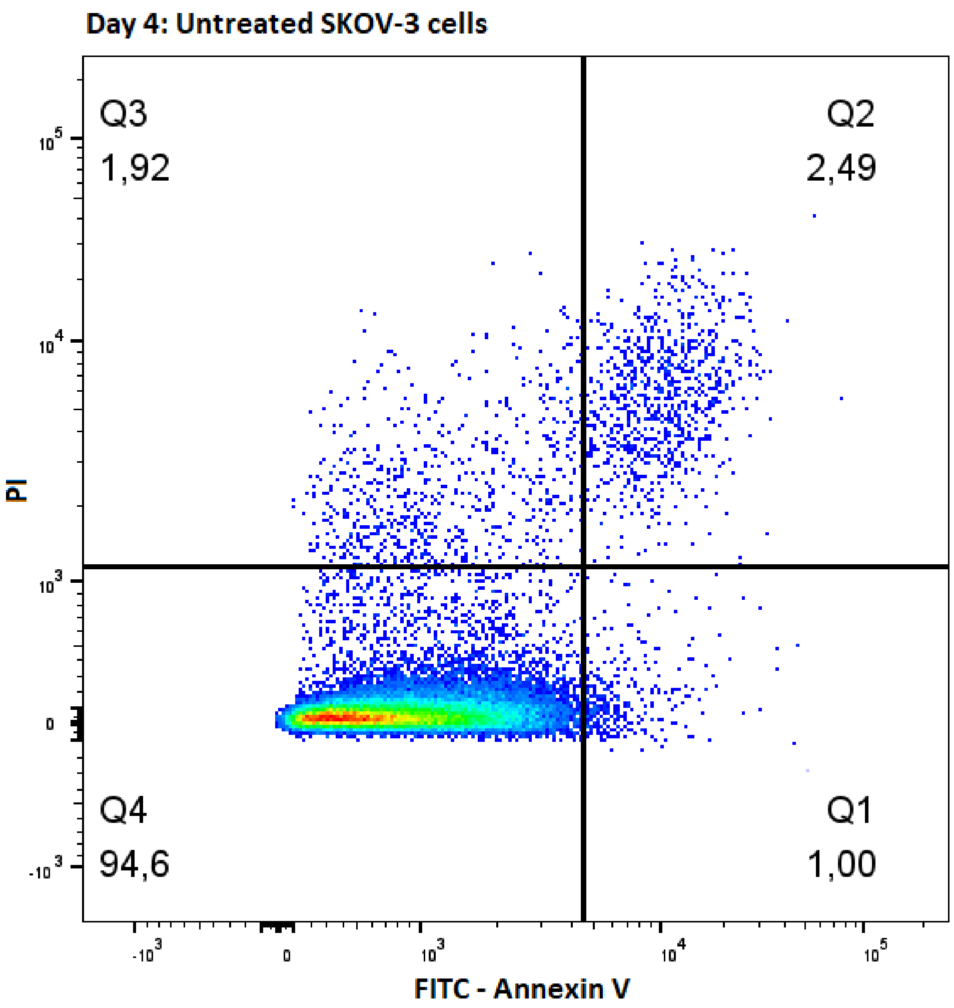


**Figure S18.** Determination of viability, apoptosis, or necrosis of untreated SKOV-3 cells at day 4. The figure is from a representative experiment performed three times with similar results. The mean ± SEM (Standard Error of the Mean) values of apoptotic cells are shown in Table 1 and Figure 7. Detailed information is provided in the legend of Figure 6.

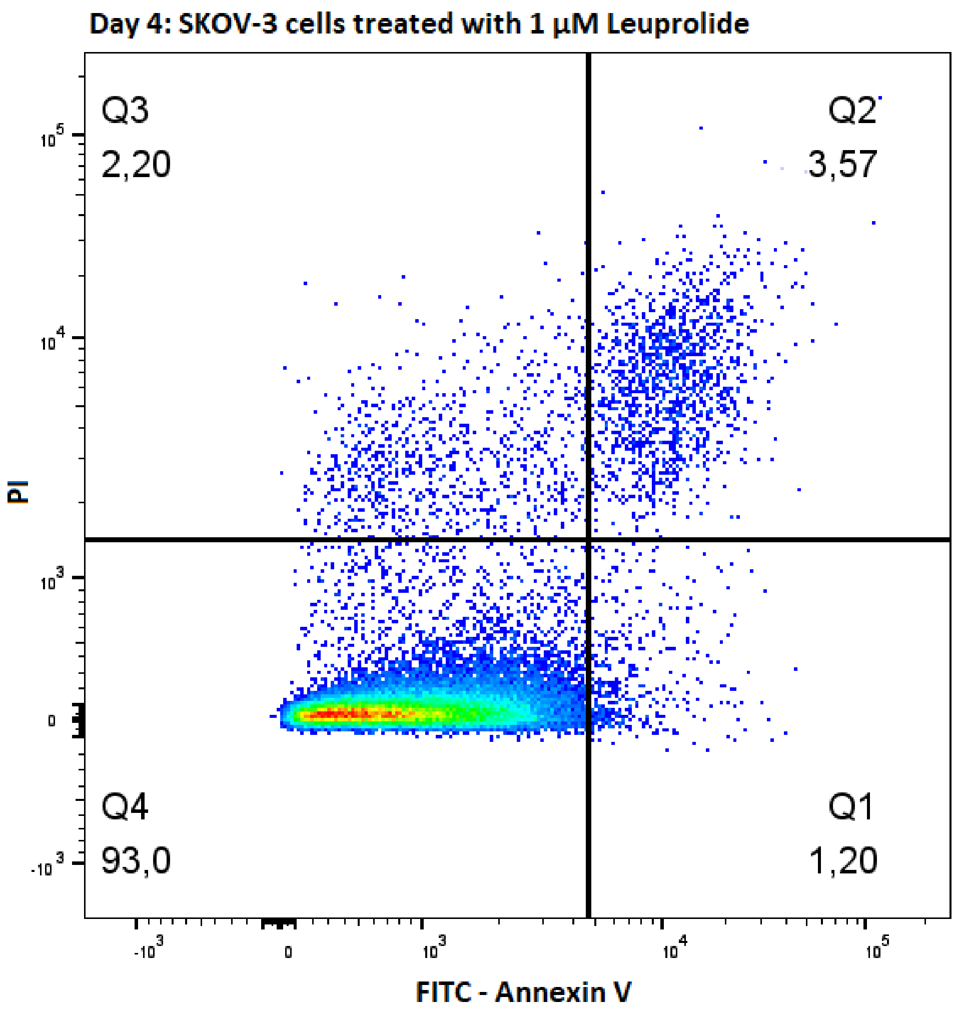


**Figure S19.** Determination of viability, apoptosis, or necrosis of SKOV-3 cells after their treatment with 1 µM leuprolide for 4 days at 37°C. The figure is from a representative experiment performed three times with similar results. The mean ± SEM (Standard Error of the Mean) values of apoptotic cells are shown in Table 1 and Figure 7. Detailed information is provided in the legend of Figure 6.

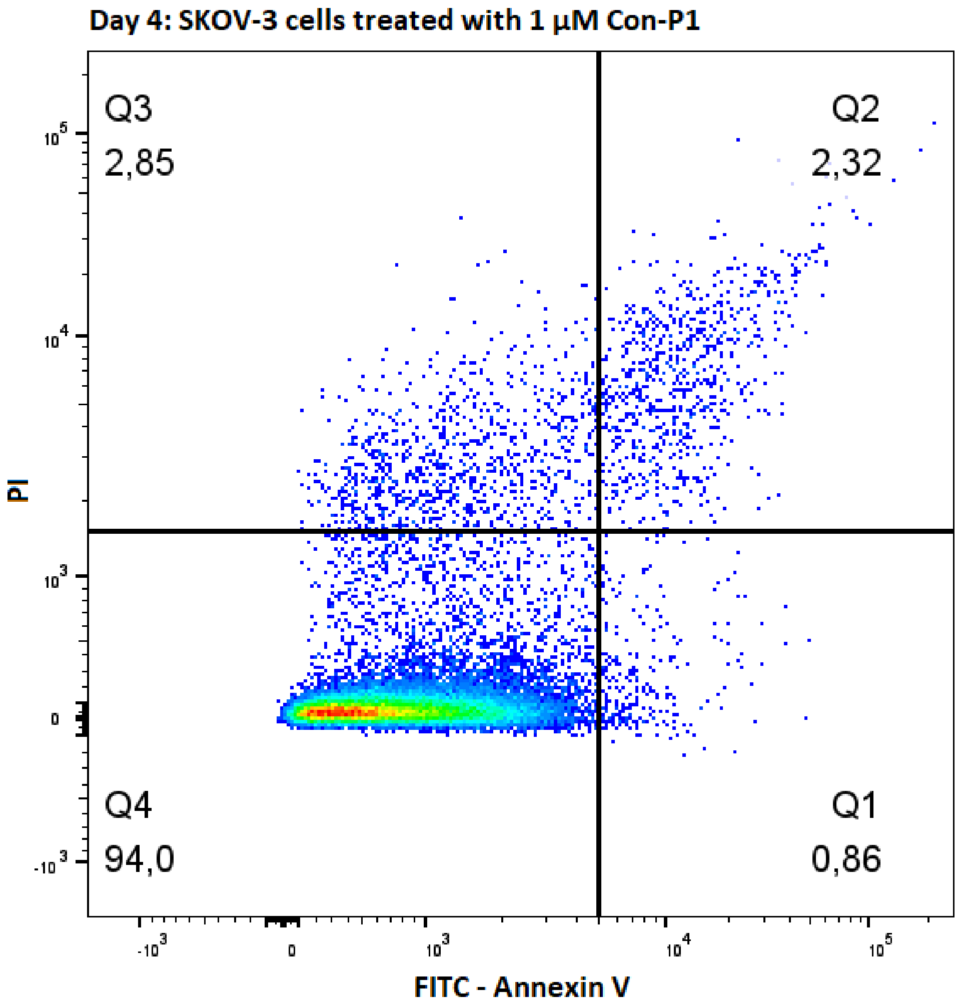


**Figure S20.** Determination of viability, apoptosis, or necrosis of SKOV-3 cells after their treatment with 1 µM Con-P1 for 4 days at 37°C. The figure is from a representative experiment performed three times with similar results. The mean ± SEM (Standard Error of the Mean) values of apoptotic cells are shown in Table 1 and Figure 7. Detailed information is provided in the legend of Figure 6.

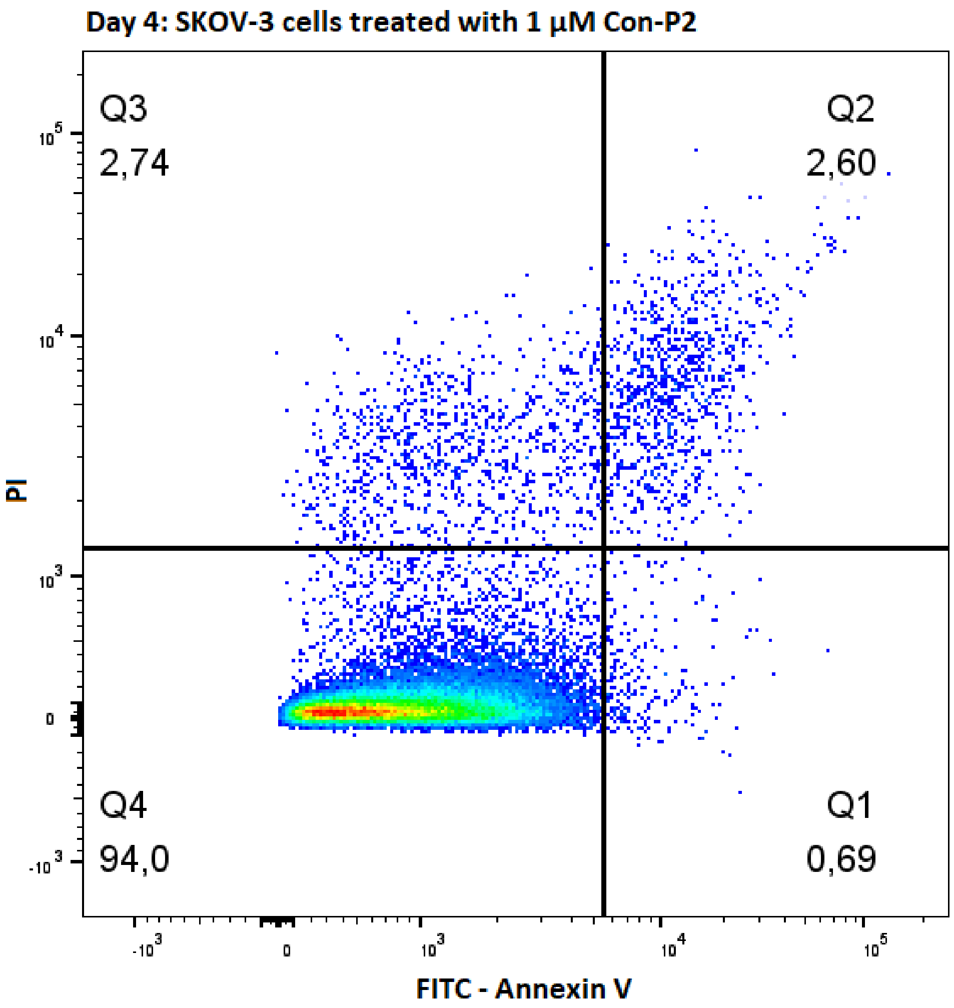


**Figure S21.** Determination of viability, apoptosis, or necrosis of SKOV-3 cells after their treatment with 1 µM Con-P2 for 4 days at 37°C. The figure is from a representative experiment performed three times with similar results. The mean ± SEM (Standard Error of the Mean) values of apoptotic cells are shown in Table 1 and Figure 7. Detailed information is provided in the legend of Figure 6.
